# Cytokinin *N*-glucosides: Occurrence, Metabolism and Biological Activities in Plants

**DOI:** 10.3390/biom11010024

**Published:** 2020-12-28

**Authors:** Eva Pokorná, Tomáš Hluska, Petr Galuszka, H. Tucker Hallmark, Petre I. Dobrev, Lenka Záveská Drábková, Tomáš Filipi, Katarína Holubová, Ondřej Plíhal, Aaron M. Rashotte, Roberta Filepová, Jiří Malbeck, Ondřej Novák, Lukáš Spíchal, Břetislav Brzobohatý, Pavel Mazura, Lenka Zahajská, Václav Motyka

**Affiliations:** 1 Laboratory of Hormonal Regulations in Plants, Institute of Experimental Botany of the Czech Academy of Sciences, Rozvojová 263, 165 02 Prague 6, Czech Republic; hluska@ueb.cas.cz (T.H.); dobrev@ueb.cas.cz (P.I.D.); l.zaveska.drabkova@ueb.cas.cz (L.Z.D.); filepova@ueb.cas.cz (R.F.); malbeck@ueb.cas.cz (J.M.); 2 Department of Biochemistry, Faculty of Science, Palacký University, Šlechtitelů 11, 783 71 Olomouc, Czech Republic; petr.galuszka@upol.cz (P.G.); katarina.mrizova@gmail.com (K.H.); 3 Department of Biological Science, Auburn University, Auburn, AL 36849, USA; hth0003@auburn.edu (H.T.H.); amr0008@auburn.edu (A.M.R.); 4 Department of Molecular Biology and Radiobiology, Faculty of AgriSciences, Mendel University in Brno, Zemědělská 1665/1, 613 00 Brno, Czech Republic; tomfi@centrum.cz (T.F.); bretislav.brzobohaty@mendelu.cz (B.B.); pavel.mazura@upol.cz (P.M.); 5 CEITEC—Central European Institute of Technology, Mendel University in Brno, Zemědělská 1, 613 00 Brno, Czech Republic; 6 Laboratory of Growth Regulators, Institute of Experimental Botany of the Czech Academy of Sciences and Faculty of Science of Palacký University, Šlechtitelů 27, 783 71 Olomouc, Czech Republic; ondrej.plihal@upol.cz (O.P.); ondrej.novak@upol.cz (O.N.); 7 Department of Chemical Biology and Genetics, Centre of the Region Haná for Biotechnological and Agricultural Research, Faculty of Science, Palacký University, Šlechtitelů 27, 783 71 Olomouc, Czech Republic; lukas.spichal@upol.cz; 8 Institute of Biophysics of the Czech Academy of Sciences, Královopolská 135, 612 65 Brno, Czech Republic; 9 Isotope Laboratory, Institute of Experimental Botany of the Czech Academy of Sciences, Vídeňská 1083, 142 20 Prague 4, Czech Republic; zahajska@ueb.cas.cz

**Keywords:** cytokinin, *N*-glucosides, senescence, β-D-glucosidase Zm-p60.1, [2-^3^H]*t*Z9G, oat, *Zea mays*, *Arabidopsis*, *trans*-zeatin, *cis*-zeatin, cytokinin oxidase/dehydrogenase

## Abstract

Cytokinins (CKs) are a class of phytohormones affecting many aspects of plant growth and development. In the complex process of CK homeostasis in plants, *N*-glucosylation represents one of the essential metabolic pathways. Its products, CK *N*7- and *N*9-glucosides, have been largely overlooked in the past as irreversible and inactive CK products lacking any relevant physiological impact. In this work, we report a widespread distribution of CK *N*-glucosides across the plant kingdom proceeding from evolutionary older to younger plants with different proportions between *N*7- and *N*9-glucosides in the total CK pool. We show dramatic changes in their profiles as well as in expression levels of the *UGT76C1* and *UGT76C2* genes during *Arabidopsis* ontogenesis. We also demonstrate specific physiological effects of CK *N*-glucosides in CK bioassays including their antisenescent activities, inhibitory effects on root development, and activation of the CK signaling pathway visualized by the CK-responsive YFP reporter line, *TCSv2::3XVENUS*. Last but not least, we present the considerable impact of CK *N*7- and *N*9-glucosides on the expression of CK-related genes in maize and their stimulatory effects on CK oxidase/dehydrogenase activity in oats. Our findings revise the apparent irreversibility and inactivity of CK *N*7- and *N*9-glucosides and indicate their involvement in CK evolution while suggesting their unique function(s) in plants.

## 1. Introduction

Cytokinins (CKs) are endogenous phytohormones acting at low concentrations as essential players in a wide variety of plant physiological processes (e.g., cell division, de novo organ formation, shoot and root development, growth of auxiliary buds, chlorophyll biosynthesis and nutrients translocation) [1,2]. The primary structural feature of CKs is the presence of *N*^6^-substituted adenine with an isoprenoid or aromatic *N*^6^-side chain that can result in dozens of different CK forms in planta. Predominant natural isoprenoid CKs are *N*^6^-(Δ^2^-isopentenyl)adenine (iP), *trans*-zeatin (*t*Z), *cis*-zeatin (*c*Z) and dihydrozeatin (DHZ). Derivatives of *N*^6^-benzyladenine (BA) represent the natural aromatic ones [3]. Both isoprenoid and aromatic CK groups substantially differ in terms of biochemical properties, receptor affinities, biological activities, and amounts in plants [4,5,6,7,8].

Modification of the CK molecule seems to predict its bioactivity. In plant tissues, CK free bases represent the active forms [9]. Attachment of ribose or glucose to the CK skeleton leads to either transport forms with no or very weak CK activity (ribosides) or reversible storage and putatively irreversible deactivation forms (*O*- and *N*-glucosides, respectively). To regulate a pool of individual CK forms and to maintain CKs at optimal active levels during plant growth, numerous CK interconversions occur in plant tissues. The most common modification of the adenine molecule is glycosylation occurring either on the purine ring at *N*3-*, N*7-, or *N*9-positions (*N*-glucosylation) or on the hydroxylated *N*^6^-side chain (*O*-glucosylation and *O*-xylosylation) [10,11,12]. These reactions are catalysed by enzymes transferring nucleotide-diphosphate-activated sugars, usually UDP-glucose.

In *Arabidopsis thaliana*, the uridine diphosphate glycosyltransferase (UGT) superfamily comprises 107 putative UGT genes and 10 UGT pseudogenes, including five UGTs with identified activity towards CKs [13,14,15]. UGT76C1 and UGT76C2 are known to form CK *N*7- and *N*9-glucosides whereas UGTs 85A1, 73C1, and 73C5 encode proteins that glucosylate *t*Z, *c*Z and DHZ at the *O*-position [15]. The role of UGTs 76C1, 76C2 and 85A1 in planta was clarified in studies using constitutive overexpressors and loss-of-function mutants [16,17,18,19,20]. In *Arabidopsis UGT76C2* knock-out plants, Lee et al. [21] reported different physiological roles of CK *N*-glucosides in roots and shoots.

CK *N*7- and *N*9-glucosides have been for years generally considered CK forms with none or very low activity in bioassays [22,23] although a few exceptions exist in older literature, mostly reported for BA *N*-glucosides [24,25,26]. Physiological activities of *t*Z *N*9-glucoside (*t*Z9G) and DHZ *N*9-glucoside (DHZ9G) were also reported on soybean callus growth by Van Staden and Drewes [27] who attributed their effects to the subsequent formation of corresponding free bases. Recently, antisenescent effects were demonstrated for *t*Z *N*7-glucoside (*t*Z7G) and *t*Z9G in *Arabidopsis* plants, together with their impact on transcriptome and proteome [28]. These authors concluded that hydrolysis of *t*Z *N*-glucosides does not contribute significantly to their antisenescent activities, as they were not able to delay senescence in mutants lacking functional copies of CYP735A1 and CYP735A2, the enzymes catalysing hydroxylation of iP-type CKs and thus the production of *t*Z-types [29]. Therefore, de novo conversion of iP to *t*Z should be required, although iP does not possess the antisenescence activity [30,31].

As no interaction of CK *N*-glucosides with the CK perception system has been revealed yet, the core of the mechanisms behind the bioactivities of *N*-glucosides in some physiological processes remains to be clarified. Whereas Hallmark et al. [28] suggested several possible mechanisms of *t*Z *N*-glucosides mode of action distinct from their hydrolysis, Hošek et al. [32] contrarily showed their immediate conversion to *t*Z in metabolic studies on *Arabidopsis* seedlings treated with *t*Z7G or *t*Z9G as well as in *Arabidopsis* cells incubated with [^3^H]*t*Z9G. In analogy, Podlešáková et al. [33] observed free bases upon treating maize seedling with *N*9-glucosides of 3-methoxy-*N*^6^-benzyladenine, BA and DHZ.

The CK activity of *N*3- and *O*-glucosides is mediated via the backward conversion to the active forms catalysed by β-D-glucosidase [3]. The β-D-glucosidase (EC 3.2.1.21) of 60 kDa (p60) was firstly isolated from maize coleoptiles [34]. Its physiological relevance was described later based on the transient and constitutive expression of Zm-p60.1 (coding for a protein with the same enzymatic properties as p60) in tobacco protoplasts utilizing CK *N*3- and *O*-glucosides but not *N*7- and *N*9-glucosides as substrates [35,36]. It has been found that Zm-p60.1 also hydrolyzes *t*Z9G although at a rate 1000-fold lower than with the *O*-glucosides and hydrolysis of *t*Z7G could not be detected at all [37]. The existence of putative enzymatic pathway (isomerization) involved in the conversion of two *t*Z *N*-glucoside isomers has been suggested by Lee et al. [21].

CKs mainly trigger physiological responses in planta through a series of transcriptional responses activated via canonical two-component signaling system typically consisting of a sensory histidine kinase (HK) and response regulators (RRs) [2]. In *Arabidopsis*, a multistep-phosphorelay system including CK receptors AHK2, AHK3, CRE1/AHK4/WOL1, histidine phosphotransfer proteins (AHP1-5 and AHP6 lacking His residue) and type-A and type-B ARRs was described [1,38,39]. Whereas *O*- and *N*-glucosylated *t*Z derivatives tested in bacterial *E. coli* assay expressing the CRE1/AHK4 and AHK3 exhibited no activity, they were found to be biologically active in *Arabidopsis* reporter gene assay. However, *t*Z7G and *t*Z9G showed only relatively low expression of the ARR5 gene in comparison with *t*Z *O*-glucoside (*t*ZOG) and *t*Z *O*-glucoside riboside (*t*ZROG) [7]. Recent results of other studies [9] support the concept that CK *N*-glucosides do not directly interact with the CK perception system.

Irreversible metabolic degradation of isoprenoid CKs in plants by removal of the *N*^6^-side chain is catalysed specifically by cytokinin oxidases/dehydrogenases (CKXs) [40,41]. Extensive studies of substrate preferences of particular CKX isozymes revealed that some recombinant *Arabidopsis* AtCKX [42,43], barley HvCKX [44] and maize ZmCKX [45] isoforms show an ability to degrade CK *N*9- but probably not CK *N*7-glucosides. Important complementation of CK down-regulation pathways is well documented in *Arabidopsis ugt76c2* plants in which decreased production of CK *N*-glucosides resulted in higher activity of CKX in roots but not in shoots [32].

The endogenous levels of CK *N*-glucosides are variable throughout the plant kingdom. Very low or trace amounts of CK *N*-glucosylated forms together with mostly rather low levels of CK *O*-glucosides were reported in non-vascular plants [46,47], which is in contrast with high concentrations of CK *N*-glucosides (with a different share of *N*7- and *N*9-forms) generally detected in vascular plants [48]. An extra- and intracellular distribution study in *Arabidopsis* and barley leaves revealed the predominant occurrence of CK *N*-glucosides in the apoplast and to a lesser extent in vacuoles. However, not in the cytosol [49].

Despite very recent progress in the research of CK *N*-glucosides in plants [21,28,32], existing knowledge about their role in the evolution and biology of CKs is very limited. In this work, we provide new information regarding occurrence, metabolism and biological activities of CK *N*7- and *N*9-glucosides to reconsider their apparent irreversibility and inactivity and to clarify their potential biological functions and significance in higher plants.

## 2. Materials and Methods

### 2.1. Arabidopsis Thaliana Ontogeny and qRT-PCR Analysis

*Arabidopsis thaliana* seeds (ecotype Columbia-0) were surface sterilized by the mixture of 1% (*v*/*v*) sodium hypochlorite and 0.02% (*v*/*v*) Triton X-100 for 10 min and rinsed 3 times with sterile water, sown on Petri dishes (12 × 12 cm) containing half-strength MS medium (1.2% agar) and stratified for 3 d at 6 °C in the dark. The plates were then transferred into the cultivation room (16 h light at 20 °C/8 h dark at 18 °C). At 3, 7, 14, 21, 35, and 49 days after sowing (DAS), the leaves and roots (approximately 100 mg of fresh weight; FW) were collected separately into 2 mL Eppendorf tubes in three replicates and frozen immediately in the liquid nitrogen.

*Arabidopsis* total RNA from leaves and roots was extracted (RNeasy Plant Mini Kit, Qiagen, Hilden, Germany) and treated with the RNase-free DNase I (Promega, Madison, WI, USA). An aliquot of 2 µg was used as a template for reverse-transcription (RevertAid™ H Minus First Strand cDNA Synthesis Kit, Fermentas, Thermo Fisher Scientific, Waltham, MA, USA) using (dT)18 following the instructions in the manual. Transcript levels of *UGT76C1* and *UGT76C2* were measured by GoTaq qPCR Master Mix (Promega) on a LightCycler 480 II (Roche, Basel, Switzerland). PCR reactions were performed in triplicate using 1 µg of cDNA and 0.2 µM of each primer with SYBR Green mix (Promega) in a final volume of 10 µL. Negative controls were included in each run. Data obtained were normalized according to Arabidopsis elongation factor 1 and actin genes. *UGT* primers for qRT-PCR were used according to previously published protocols [16,18].

### 2.2. Maize Phenotype Traits and qRT-PCR Analysis

Maize seeds (*Zea mays* L. cv. Cellux) were imbibed in tap water and germinated for 3 d on a wetted filter paper. Germinated seedlings were transferred to aerated hydroponic tanks filled with Hoagland nutrient solution. CKs (*t*Z7G and *t*Z9G) diluted in DMSO were added at 0.1 µM and 5 µM into the solution after seedlings were nested for 3 days. The controls contained the corresponding amount of DMSO (0.01%) in the hydroponic solution. Plants were cultivated for 10 d in a growth chamber (16 h light at 27 °C/8 h dark at 20 °C). Length of primary roots and primary leaves of 15 plants were measured with a ruler. The experiment was repeated twice in three biological replicates.

Expression profiling of genes coding for maize *IPTs* (*IPT5* and *IPT6*), *CKXs* (*CKX1* and *CKX4b*), and *RRs* (*RR1, RR6* and *RR7*), including primer sequences and data evaluation, was performed as described by Vyroubalová et al. [50]. Total RNA for reverse transcription was isolated from homogenized plant tissues (100 mg FW) using the RNAqueous kit and Plant RNA Isolation Aid solutions (Ambion, Thermo Fisher Scientific, Waltham, MA, USA), treated twice by TURBO DNA-free^TM^ kit (Life Technology, Thermo Fisher Scientific, Waltham, MA, USA) to remove all traces of genomic DNA contamination and used for first-strand cDNA synthesis by RevertAid^TM^ H Minus M-MuLV RT and oligo-dT (Fermentas). Diluted cDNA samples were used as templates in qRT-PCR reactions containing POWER SYBR^®^ Green PCR Master mix or Taq Man^®^ Gene Expression Master Mix (Life Technology), 300 nM of each primer and 250 nM specific 5′-FAM and 3′-NFQ labeled MGB or standard Taq Man probe, respectively. RNA from three biological replicates were transcribed in two independent reactions, and each of the cDNA samples was run in at least two technical replications on StepOnePlus^TM^ Real-Time PCR System (Life Technology) in a default program. C_t_ values were normalized for β-actin and elongation factor 1 genes. Expression values were determined in maize leaves and roots at 5 h, 24 h and 72 h after applications of *t*Z, *t*Z7G and *t*Z9G (0.5 µM and 5 µM) and statistically evaluated with DataAssist v3.0 Software (Life Technology). The Benjamini and Hochberg false discovery rate was used to obtain adjusted *p*-values [51] for the unpaired Student’s *t*-test (* *p* < 0.05).

### 2.3. Chlorophyll Retention Bioassay

Oat (*Avena sativa* L. cv. Abel) seeds were soaked overnight in distilled water and sown into perlite saturated with 2-fold concentrated modified Knop’s nutrient solution (6.1 mM Ca(NO_3_)_2_x.4H_2_O, 1.8 mM KH_2_PO_4_, 60 µL 5% FeCl_3_.6H_2_O (*v*/*v*), 2.1 mM MgSO_4_x7H_2_O and 1.6 mM KCl), pH adjusted to 5.7 with NaOH). Plants were cultivated for 10 d in a growth chamber (SANYO MLR 350H; Sanyo, Japan) under controlled long-day conditions (18 h light at 20 °C/6 h dark at 18 °C). First leaves were cut to 7 cm long apical segments. The basal ends of four segments were placed into 15 mL polystyrene tubes filled with 1 mL of tested CK solutions (*t*Z, *t*Z7G, *t*Z9G, iP, iP7G, iP9G, DHZ7G or DHZ9G; all OlChemIm, Czech Republic) applied at concentrations 0.32 nM; 0.16 µM; 0.8 µM; 4 µM; 0.02 mM, 0.1 mM or 0.5 mM or water and incubated in darkness for 4 d at 26 °C. For assessment of endogenous CK profiles, oat leaf segments incubated for 4 d in darkness at 26 °C with 0.5 mM solution of *trans*-zeatin, N^6^-(Δ^2^-isopentenyl)adenine and their *N*7- and *N*9-glucosides were used.

Chlorophyll was extracted by 10 mL 80% ethanol (*v*/*v*) at 80 °C for 15 min according to a slightly modified procedure by Kamínek et al. [52]. The optical absorbance of leaf extracts was measured on a spectrophotometer (Helios Alpha; Thermo Fisher Scientific, USA) at wavelengths 643.5 nm and 661 nm. Chlorophyll retention was expressed as absorbance/fresh weight ratio percentage of fresh oat leaf segments. Extracts from non-incubated fresh oat leaves are presented as C1“initial“ control and as C2 control from water-treated leaf segments. Experiments were repeated twice with at least three biological replicates.

### 2.4. Metabolism of Tritium Labeled Trans-Zeatin 9-Glucoside in Oat Leaf Segments

Tritium labeled [2-^3^H]*t*Z9G was applied to oat leaf segments submerged into 1 mL incubation solution containing 0.658 µM of [2-^3^H]*t*Z9G (0.7716 MBq) completed with 0.1 mM unlabeled *t*Z9G. After 4 d incubation under the same conditions as for chlorophyll retention bioassay (see above), the oat leaf segments were frozen in liquid nitrogen. Before extraction of CKs, the samples were ground by FastPrep-24^TM^ 5G grinder at speed 6.0 m·s^−1^ for 40 s using AllMetalQuickprep adapter (MP Biomedicals, Irvine, CA, USA). The metabolism of radiolabeled CKs was analyzed on an HPLC Ultimate 3000 (Thermo Fisher Scientific, Waltham, MA, USA) coupled to a radioactivity-HPLC flow detector (Ramona 2000; Raytest, Straubenhardt, Germany) with on-line admixture at volumetric ratio 3:1 of scintillation cocktail Flo-Scint II (Perkin Elmer, Waltham, MA, USA). The analysis was done on a Kinetex C18 HPLC column (5 µm, 150 mm × 4.6 mm; Phenomenex, Torrance, CA, USA) at 0.6 mL/min with a tertiary gradient of A: 400 mM ammonium acetate, pH 4, B: 5% methanol in water by volume, and C: methanol/acetonitrile (1:1, *v*/*v*). The gradient programme was 5–15% C for 10 min, 15–34% C for 14 min, 100% C for 1 min, with A kept constant at 5%. Metabolite identification was based on comparison of retention times of applied standards.

### 2.5. Senescence Bioassay in Tomato

For the CK senescence bioassay, tomato leaf discs (7 mm diameter) cut from 60 d old MicroTom fully expanded leaves grown in a growth chamber (16 h light at 26 °C/8 h dark at 22 °C), were immediately transferred to Petri dishes (5 cm diameter) containing MES buffer with dimethyl sulfoxide (DMSO; control, pH 5.7 by KOH) or 5 μM of different CKs (*t*Z, *t*Z7G, or *t*Z9G) diluted in DMSO. After 6 d incubation in darkness, the tomato leaf discs (*n* = 6, three discs per replicate) were washed in MES buffer and briefly dried before measuring the absorbance at 652 nm, 665 nm and 470 nm wavelengths to quantify the contents of photosynthetic pigments (Chl *a*, Chl *b,* and carotenoids). The pigment quantity was expressed as a measure of μg·mL^−1^ according to Sumanta et al. [53].

### 2.6. Analysis of Endogenous Cytokinins in Plant Tissues

Endogenous CKs were extracted from homogenized plant tissue (approximately 100 mg FW) using 0.5 mL of cold Bielski buffer consisting of methanol/formic acid/water (15/1/4, *v*/*v*/*v*) according to a published method [54]. Following the addition of stable isotope labeled internal standards ([^2^H_5_]*t*Z, [^2^H_5_]*t*ZR, [^2^H_5_]*t*Z7G, [^2^H_5_]*t*Z9G, [^2^H_5_]*t*ZOG, [^2^H_5_]*t*ZROG, [^2^H_5_]*t*ZRMP, [^2^H_3_]DHZ, [^2^H_3_]DHZR, [^2^H_3_]DHZ9G, [^2^H_6_]iP, [^2^H_6_]iPR, [^2^H_6_]iP7G, [^2^H_6_]iP9G, [^2^H_6_]iPRMP; 10 pmols each), samples were extracted for 1 h at −20 °C. The solids were separated by centrifugation (20,000× *g*, 25 min, 4 °C). The pellets were re-extracted with an additional 0.5 mL of cold Bielski buffer for 30 min at 6 °C. Samples were concentrated in a vacuum concentrator (Alpha RVC, Christ GmbH, Osterode am Harz, Germany), diluted with 0.5 mL of 1 M formic acid and applied to a mixed-mode reversed phase-cation exchange SPE column (Oasis-MCX, Waters, Milford, MA, USA). The columns were washed with water and 100% methanol and CK fraction was sequentially eluted with 0.35 M NH_4_OH in 60% methanol. The fraction was evaporated to dryness in a vacuum concentrator and dissolved in 5% methanol. Cytokinin quantification was done on an HPLC Ultimate 3000 (Dionex, Bannockburn, IL, USA) coupled to a hybrid triple quadrupole/linear ion trap mass spectrometer (3200 Q TRAP, Applied Biosystems, Thermo Fisher Scientific, Waltham, MA, USA) using a multilevel calibration graph with [^2^H]-labeled internal standards as described before [55,56]. CK analysis in selected plant species and during Arabidopsis ontogeny were performed according to Gajdošová et al. [48].

Chromatographic conditions included an HPLC column Luna C18(2) (150 mm × 2 mm, 3 µm, Phenomenex, Torrance, CA, USA) kept at 35 °C, a flow rate of 0.25 mL/min, and a linear gradient of solvent A (5 mM ammonium acetate, pH 4 in water) and solvent B (5 mM ammonium acetate, pH 4, in methanol) from 10 to 40% B for 20 min. The mass spectrometer was run in positive electrospray ionization mode. Ion source parameters included ion source voltage of 4500 V, nebulizer gas at 50 psi, heater gas at 60 psi, curtain gas at 20 psi, and heater gas temperature of 500 °C. Quantification of hormones was done using the isotope dilution method with multilevel calibration curves (r^2^ > 0.99). Data processing was carried out with Analyst 1.5 software (Applied Biosystems, Foster City, CA, USA).

The radiolabeled CK metabolites were extracted from oat leaf segments (approximately 100 mg FW) according to the protocol described above, but omitting the addition of internal standards. Besides plant tissues, incubation solutions after 4 d incubation were also examined. All samples were analyzed by passing the HPLC eluate through a radioactivity flow detector (Ramona 2000; Raytest GmbH, Straubenhardt, Germany) coupled to the HPLC Ultimate 3000 chromatograph (for more details see Section 2.4). All [^3^H]CKs used in the bioassay ([2-^3^H]iP, [2-^3^H]*t*Z, and [2-^3^H]*t*Z9G) were synthesized as previously reported for *N*^6^-alkyladenosines labeled with tritium [57].

### 2.7. Cytokinin Oxidase/Dehydrogenase Activity and Substrate Specificity

To determine CKX activity, 14 d old oat leaf segments were incubated for 4 d under same conditions as for the chlorophyll retention bioassay (see above) in water or tested CK solutions (*t*Z, *t*Z7G, *t*Z9G, iP, iP7G and iP9G) applied at 0.1 mM and 0.5 mM concentrations. Proteins were extracted and CKX partially purified according to Motyka et al. [58]. The CKX activity was determined by an in vitro radioisotope assay based on the conversion of tritium-labeled iP to adenine. The assay mixture (50 µL final volume) contained 100 mM TAPS-NaOH buffer (pH 8.5) including 75 µM 2,6-dichloroindophenol, 2 µM [2-^3^H]iP and the enzyme preparation equivalent to 4 mg FW tissue. After incubation at 37 °C for 2 h, the reaction was terminated by adding 10 µL 200 mM Na_4_EDTA and 120 µL 95% (*v*/*v*) ethanol, and the substrate and the product were separated using HPLC as described elsewhere [59]. Protein concentrations were determined according to Bradford [60] using bovine serum albumin as a standard.

The substrate specificity of CKX was determined based on the competitive effects of unlabeled CKs (2 µM and 20 µM) on the in vitro degradation of 2 µM [2-^3^H]iP as described by Motyka and Kamínek [61,62].

### 2.8. Metabolic Stability of Trans-Zeatin N7- and N9-Glucosides in Tested Stock Solutions

The metabolic stability of *t*Z7G and *t*Z9G in tested solutions prepared for chlorophyll retention bioassay was assessed at the beginning of the experiment (time 0; i.e., fresh stocks) and after 4 d incubation in darkness (with or without leaf segments). Concentrations of CKs were measured in 5 µL injected volume of 0.5 nmol·mL^−1^ *t*Z7G and *t*Z9G solutions by HPLC/MS system consisting of HTS-Pal auto-sampler with a cooled sample stack (CTC Analytics AG, Zwingen, Switzerland), a quaternary HPLC pump Rheos 2200 (Flux Instruments AG, Reinach, Switzerland), a DeltaChrom CTC 100 column oven (Watrex Ltd., Prague, Czech Republic) and a TSQ Quantum Ultra AM triple-quadrupole mass spectrometer (Thermo Electron, Thermo Fisher Scientific, Waltham, MA, USA) equipped with an electrospray interface. The HPLC column Synergy Hydro-RP (250 × 2 mm, 4 µm, Phenomenex, Torrance, CA, USA) was used to separate the analytes at mobile phase flow rate of 0.2 mL·min-1 and column oven temperature 40 °C. The mobile phase consisted of aqua (A), acetonitrile (B) and 0.01% acetic acid (C). The ternary gradient started at 7.5% of solvent A for the first 10 min, then increased to 22% for 10 min and to 50% during the next 16 min. The proportion of solvent C was maintained at 30% throughout the analysis. The mass spectrometer was operated in positive MS/MS mode with interface settings: spray voltage 4500 V, sheath gas 60 units and auxiliary gas 40 units. For each precursor, the most intense fragment was recorded for quantitation and 2–3 more to confirm identity.

CKs were quantified using multilevel calibration graphs with [^2^H]-labeled internal standards used, and their contents were expressed as pmol·mL^−1^. The calibration allowed quantification at a range of 0.1 to 1000 pmol·mL^−1^. For each variant, two independently incubated samples were analyzed.

### 2.9. β-D-Glucosidase Activity Assay

A native protein of β-D-glucosidase was extracted from frozen oat leaf segments used in chlorophyll retention assay. Samples (500 mg FW) were homogenized in liquid nitrogen and then transferred to a testing tube containing 2 mL of extraction buffer consisting of 100 mM Tris-HCl (pH 8.5), 50 mM NaCl, 2 mM MgCl_2_·6H_2_O, 0.05% sodium dodecyl sulphate (SDS) and 1 mM phenylmethylsulfonyl fluoride (PMSF). Protein extraction was carried out for 30 min at 25 °C. The solution was subsequently centrifuged (30,000× *g*, 30 min, 4 °C), and the clear supernatant was immediately transferred to pre-chilled testing tube and stored on ice. Extract isolated from oat leaves containing 10 μg of native protein was added to *t*Z9G or *t*ZOG dissolved in 0.05 M citrate-phosphate buffer McIlvaine (pH 5.5) to a final concentration 9 mM with subsequent incubation for 0 h, 24 h, 48 h, 72 h or 168 h at 30 °C. Visualization of products formed by enzymatic hydrolysis of the substrates was then performed based on thin-layer chromatography method (TLC). The reaction mixture (2 µL) was directly loaded onto Pre-coated SIL G-25 UV_254 + 366_ TLC plates (silica gel with UV indicator, layer thickness 0.25 mm; Machery-Nagel GmbH, Dueren, Germany) and separated using *n*-butanol/acetic acid/ddH_2_O (12/3/5, *v*/*v*/*v*) as mobile phase. Plates were developed for 5 h at 25 °C. Spots were visualized with UV transilluminator at 254 nm, and digital images of the thin-layer chromatograms were recorded by the camera (Olympus SP-350) in RAW format and cropped in Adobe Photoshop CS6 without altering original image quality and integrity. The data were obtained from two biological replicates, including two different bioassay replicates.

### 2.10. Cytokinin Receptor Assays

For *Arabidopsis* receptor measurements, constructs of *pPINIII*-*AHK4* and *pSTV28*-*AHK3* were utilized [63,64]. Full-length coding sequences of maize receptors *ZmHK1* and *ZmHK3* were obtained by gene synthesis (GeneArt, Invitrogen, Carlsbad, CA, USA) and cloned into *pPINIII* vector [33].

Bacterial activation assay in *E. coli* strain KMI001 (∆*rcsC, cps::lacZ*) was performed as described previously by Spíchal et al. [7]. The expression of the CK receptors was induced for 16 h with 25 μM isopropyl β-D-thiogalactopyranoside (IPTG) for ZmHK3 and 250 μM IPTG for ZmHK1. The cells were incubated in the presence of either *t*Z7G or *t*Z9G solution in the range of 0.64 nM to 50 µM concentration and activity of β-D-galactosidase was assayed using 4-methylumbelliferyl *O*-β-D-galactopyranoside as a substrate. The obtained data are from a representative experiment, including three replicates.

The binding assay with [2-^3^H]*t*Z was performed as described previously [65]. Briefly, overnight bacterial suspension of KMI001 harbouring *AHK* or *ZmHK* constructs was aliquoted (1.0 mL) and mixed with [2-^3^H]*t*Z (3 pmol) with or without unlabeled *t*Z7G and *t*Z9G at selected concentrations (10 µM, 1 µM, 0.1 µM and 10 nM). For assessment of total and non-specific binding of the studied receptors, bacterial suspensions were mixed with [2-^3^H]*t*Z alone or with 5000-fold excess of unlabeled *t*Z, respectively. Negative control was performed using adenine as a ligand. Samples were incubated 30 min at 4 °C and quickly centrifuged. The pellets were suspended in 1 mL of ACS-II scintillation cocktail (Amersham BioSciences UK Ltd., Buckinghamshire, UK) by successive vortexing and sonication steps. The radioactivity was measured on a scintillation counter Beckman LS 6500 (Beckman Coulter Inc., Indianapolis, IN, USA). The obtained data are from a representative experiment, including three replicates.

### 2.11. TCSv2::3XVENUS Response to Trans-Zeatin and Its N7- and N9-Glucosides in Tomato Roots

Stable CK-responsive yellow fluorescent protein YFP reporter line, *TCSv2::3XVENUS*, in tomato (*Solanum lycopersicum* L. *cv*. M82) seedlings, a generous gift from Dr. Yuval Eshed [66] was used. Plants were grown in a growth chamber (16 h light at 26 °C/8 h dark at 22 °C). The seedlings (15 d old) were treated for 24 h with MES (buffer control, pH 5.7 by KOH) or 5 μM solutions of *t*Z, *t*Z7G or *t*Z9G as described in Keshishian et al. [67]. After treatment, the plants were examined for YFP fluorescence in the primary root, mainly at the root tip and approximately at 1 cm distance from the root tip, using a Nikon Eclipse 80i epifluorescence microscope with a UV source and a YFP filter. A representative photo of a root from each treatment was taken with a Qimaging Fast 1394 digital camera and cropped using Adobe Photoshop CS3 without altering the original photo integrity.

## 3. Results

### 3.1. Occurrence and Distribution of Cytokinin N-Glucosides Throughout the Plant Kingdom

In order to obtain a general overview of the occurrence and distribution of CK glucoconjugates in land plants, endogenous contents and concentration ratios of CK *N*7- and *N*9-glucosides in fully developed vegetative organs (mostly leaves and shoots) of one hundred representative species including Lycophytes (Horsetails and Ferns), Gymnosperms (Ginkgo and Palmferns, Conifers), Angiosperms (Basal angiosperms and Magnoliids), Monocots, and Eudicots (Fabids, Malvids, Superasterids, Lamiids and Campanulids) were determined. The list of selected species together with the levels of individual CK *N*-glucosides are shown in Supplemental Table S1.

**Figure 1:**
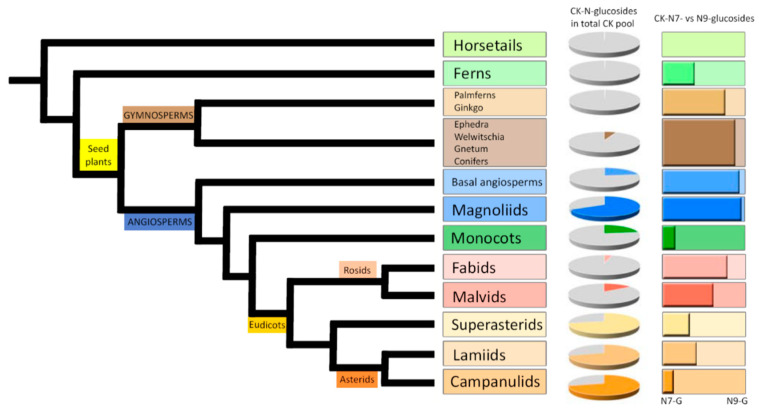
Schematic overview of the occurrence of cytokinin (CK) *N*-glucosides in the total CK pool (expressed as a percentage of total CKs) and the proportional distribution between CK *N*7- and *N*9-glucosides throughout the plant kingdom. A simplified phylogenetic tree of green plants was modified according to Byng et al. [68].

The comprehensive screen revealed that both *N*7- and *N*9-glucosides occur throughout the plant kingdom in concentrations ranging from zero to hundreds of picomols per gram of tissue FW. In evolutionarily older plants (Horsetails, Ferns, Ginkgo and Palmferns), the contents of individual CK *N*-glucosides were close to the detection limit and in total did not exceed 0.5 pmol·g^−1^ FW. Higher concentrations of total CK *N*-glucosides were found in Angiosperms, and an upward trend was observed, resulting in concentration maxima of total CK *N*-glucosides in youngest clades represented by Eudicots: Superasterids, Campanulids and Lamiids (Figure 1, Supplemental Table S1). However, even among evolutionary younger Angiosperms some of the examined species (especially Monocots, Fabids and Malvids) were found to contain the CK *N*-glucosides below the limit of detection or in minute quantities (Supplemental Figure S1A–C and Table S1). Contrary, the majority of examined species contained substantial amounts of CK *O*-glucosides (Supplemental Figure S1D and Table S1).

Varying proportions of CK *N*-glucosides (both *N*7- and *N*9-) in the total CK pool as well as the different distribution between CK *N*7- and *N*9-glucosides in selected species across the plant kingdom are demonstrated in Figure 1 and Supplemental Figure S1. Different ratios of CK *N*7- and *N*9-glucoside concentrations point to a predominance of CK *N*7-glucosides over *N*9-glucosides in Gymnosperms, Basal angiosperms, Magnoliids and Rosids (Fabids and Malvids). On the other hand, the prevalence of CK *N*9-glucosides was found mainly in Monocots, Superasterids, and Asterids (Lamiids and Campanulids). CK *N*9-glucosides were shown to prevail over *N*7-glucosides also in Ferns and Horsetails. The content of total CK *N*-glucosides in these clades was very low though (Supplemental Table S1). Interestingly, most of the examined species contained exclusively one isomer (represented by the sum of CK *N*7-glucosides or *N*9-glucosides) or the other isomer was present in minuscule quantities with only several species containing both isomers in comparable amounts (ranging from 15% to 85% from total cytokinin *N*-glucosides).

Results for the selected set of plant species entitle us to assume that the levels of total CK *N*-glucosides generally increase from lower (evolutionary older) to higher (evolutionary younger) plants. The distribution between CK *N*7- and *N*9-glucosides suggests that the ratio between the two may be due to differences in life strategy rather than evolutionary complexity.

### 3.2. Profiles of Cytokinin N-Glucosides and Expression of UGT76C1 and UGT76C2 Genes during Arabidopsis Ontogenesis

Further attention was focused on the distribution of CK *N*-glucosides together with the expression of *UGT76C1* and *UGT76C2* genes in tissues throughout plant ontogenesis using *Arabidopsis* as a model plant. Endogenous concentrations of CKs as well as the expression patterns of *UGT76C1* and *UGT76C2* genes (encoding specific UGTs involved in CK *N*7- and *N*9-glucoside formation) were measured in *Arabidopsis* leaves and roots 3, 7, 14, 21, 35 and 49 days after sowing (DAS).

**Figure 2:**
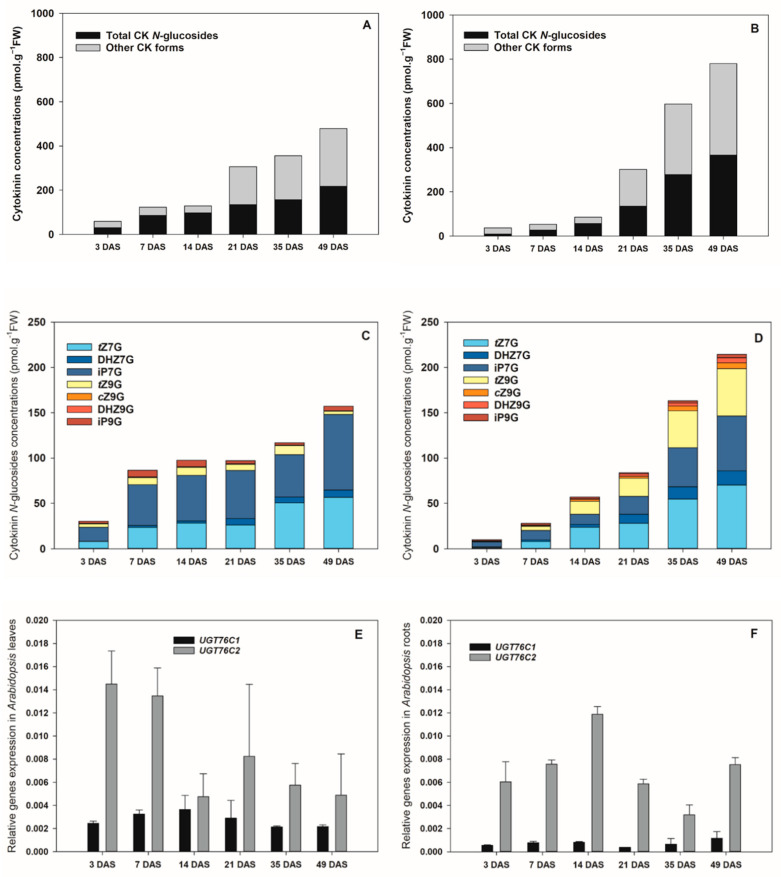
The concentration of cytokinins (CKs), distribution of CK *N*7- and *N*9-glucosides and expression patterns of *UGT76C1* and *UGT76C2* in leaves and roots throughout the ontogenesis of *Arabidopsis*. (**A**,**B**) Dynamics of endogenous contents of total CK *N*-glucosides in the total CK pool in leaves (**A**) and roots (**B**) during the *Arabidopsis* lifespan. (**C**,**D**) Distribution and dynamics of individual CK *N*7- and *N*9-glucosides in the total CK pool in leaves (**C**) and roots (**D**) during the *Arabidopsis* lifespan. (**E**,**F**) Dynamics of expression patterns of *UGT76C1* and *UGT76C2* genes in leaves (**E**) and roots (**F**) during the *Arabidopsis* lifespan. DAS = days after sowing; *t*Z = *trans*-zeatin; DHZ = dihydrozeatin; iP = *N*^6^-(Δ^2^-isopentenyl)adenine; *c*Z = *cis*-zeatin; -7G = *N*7-glucoside; -9G = *N*9-glucoside. Data are presented as mean ± standard error (SE) of three biological replicates. SE averaged 13.15% and did not exceed 32% of the means (**A**–**D**).

The HPLC/MS analysis revealed that CK *N*-glucosides, primarily those glucosylated at the *N*7-position, represent predominant CK forms in both *Arabidopsis* leaves and roots (Figure 2A–D, Supplemental Table S2). Within the *Arabidopsis* life span, a progressive accumulation of both CK *N*7- and *N*9-glucosides was recorded in leaves (Figure 2A), mainly due to the gradually increasing levels of iP7G and *t*Z7G reaching up to 83 and 56 pmol·g^−1^ FW, respectively, at 49 DAS (Figure 2C). The presence of other CK *N*7- and *N*9-glucosides in *Arabidopsis* leaves was revealed as well. Among them, especially DHZ7G, *t*Z9G, and iP9G occurred in relatively high amounts. Concentrations of none of them, however, exceeded 10 pmol·g^−1^ FW (Figure 2C, Supplemental Table S2).

Similarly to leaves, the total pool of endogenous CK *N*-glucosides in roots increased significantly over the *Arabidopsis* lifespan (Figure 2B). In roots, the levels of total CK *N*-glucosides were considerably lower than in leaves at the earlier ontogenetic stages (3–14 DAS), reached comparable values at 21 DAS and substantially exceeded the leaf concentrations at the later stages (35–49 DAS) (Figure 2A–D, Supplemental Table S2). The predominant CK *N*-glucoside forms were also represented by *t*Z7G and iP7G being followed by *t*Z9G and DHZ7G (Figure 2D, Supplemental Table S2). Other *N*-glucosides were also found in both leaves and roots, however, they occurred in considerably lower concentrations and represented only a minor proportion in both plant organs during the *Arabidopsis* ontogenesis (Figure 2C,D, Supplemental Table S2).

The analyses of the CK *N*-glucoside levels in the course of *Arabidopsis* ontogeny were supplemented by determinations of the tissue expression of *UGT76C1* and *UGT76C2* by qRT-PCR in leaves and roots. Higher expression of *UGT76C2* in comparison to the *UGT76C1* transcripts was found in both organs (Figure 2E,F). In leaves, the highest *UGT76C2* expression was revealed as early as 3 DAS and exhibited a gradually decreasing tendency at the later stages (Figure 2E). In contrast, its transcript level maximum in roots was detected at 14 DAS (Figure 2F). As for the *UGT76C1*, the highest expressions were recorded at 14 DAS in leaves and at 49 DAS in roots (Figure 2E,F).

Taken together, our data suggest that iP7G, *t*Z7G and *t*Z9G represent a substantial portion of CK *N*-glucosides in *Arabidopsis* leaves and roots and considerably participate in the total CK pool during ontogeny. The *UGT76C2* exhibits higher expression in both plant organs compared to the *UGT76C1*.

### 3.3. N9-Glucosides but not N7-Glucosides of Trans-Zeatin and Dihydrozeatin Delay Dark-Induced Senescence in Oat Leaf Segments

In order to specify their potential biological relevance, activities of *N*7- and *N*9-glucosides of *t*Z, DHZ and iP were determined in CK bioassay based on retention of chlorophyll in excised oat leaf segments and compared with those of corresponding free bases and water.

**Figure 3:**
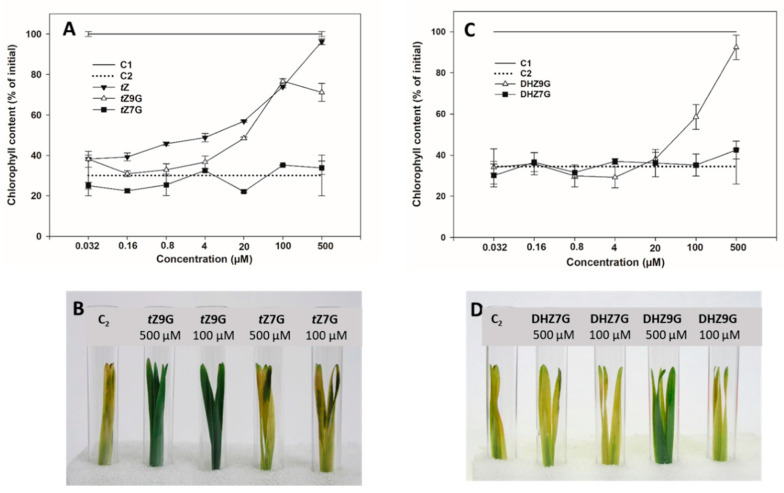
Effect of *trans*-zeatin, dihydrozeatin and their *N*7- and *N*9-glucosides on retention of chlorophyll in senescing oat leaves. Chlorophyll concentration is expressed as a percentage of the initial chlorophyll content of control fresh leaves before incubation (C1). Excised oat leaf pieces were incubated for 4 days in the dark with water (water control, C2) or solutions of cytokinins *t*Z, *t*Z7G and *t*Z9G (**A**,**B**) or DHZ7G and DHZ9G (**C**,**D**). *t*Z = *trans*-zeatin; DHZ = dihydrozeatin; -7G = *N*7-glucoside; -9G = *N*9-glucoside. Data are presented as mean ± SE of three biological replicates.

Increasing concentration of applied *t*Z resulted in a gradual increase of retained chlorophyll in oat that reached its maximum at 0.5 mM representing 96% of the initial control (C1). A similar effect on delaying chlorophyll degradation was also found for *t*Z9G (Figure 3A,B) reaching 77% of levels of C1 at its peak (0.1 mM), which corresponded to the amount as at the same concentration of *t*Z itself. In contrast to *t*Z, further increases in *t*Z9G concentration to 0.5 mM led, however, to a decline of chlorophyll content (to 71% C1). On the other hand, *t*Z7G used at the same concentrations did not cause any inhibition of the dark-induced chlorophyll degradation (Figure 3A,B). In analogy to *t*Z9G, DHZ9G also delayed chlorophyll degradation in oat leaves, at least at high concentrations (37% and 89% C1 at 0.1 mM and 0.5 mM, respectively). No antisenescent effects were found for DHZ7G (Figure 3C,D). Interestingly, no inhibition of the dark-induced senescence was recorded for iP and its *N*7- and *N*9-glucosides (Supplemental Figure S2).

In order to exclude a possibility that the antisenescent effect of *t*Z9G might be due to traces of *t*Z or any other CK constituents, both *t*Z7G and *t*Z9G stock solutions were analyzed by HPLC/MS. Moreover, to rule out potential metabolic conversions of *t*Z7G and *t*Z9G in tested solutions during incubation, the CK content was determined in freshly diluted solutions as well as in those incubated for 4 d in darkness with or without oat leaf segments. The analyses did not reveal any significant presence of other CK derivatives in the analyzed solutions, even though trace amounts (not exceeding 0.52% and 0.18% *t*Z7G and *t*Z9G, respectively) of a few CK derivatives (*c*Z, *t*Z, DHZ, and iP7G) were detected in the analyzed samples (Supplemental Table S3).

In addition, we examined whether the antisenescent effect of *t*Z9G may originate from its cleavage by β-D-glucosidase. Crude protein extracts from oat leaf segments treated with *t*Z9G (4 µM to 0.5 mM) were used to determine the activity of β-D-glucosidase towards two natural substrates, *t*Z9G and *t*ZOG applied at 9 mM concentration. The TLC chromatograms of reactions incubated for up to 7 days with *t*ZOG and *t*Z9G are shown in Supplemental Figure S4A,B, respectively. In addition to both native substrates, three artificial ones, namely chromogenic 4-nitrophenyl-β-D-glucopyranoside (pNPG*p*), 5-bromo-4-chloro-3-indolyl β-D-glucopyranoside (X-Glu*p*), fluorogenic 4-methylumbelliferyl *O*-β-D-glucopyranoside (4-MUG*p*), were tested for β-D-glucosidase activity. The β-D-glucosidase activity was detected in all tested protein extracts from oat leaf segments being able to hydrolyze two artificial substrates mentioned above (pNPG*p* and 4-MUG*p*) as well as natural one (*t*ZOG). However, no activity of β-D-glucosidase towards artificial X-Glu*p* and natural substrate *t*Z9G was revealed (data not shown).

To summarize, *t*Z9G and DHZ9G, but neither their corresponding *N*7-glucosylated counterparts nor iP and its *N*-glucosides, were found to delay dark-induced senescence in oat leaf segments. As the activity of β-D-glucosidase towards *t*Z9G as well as the contribution of other CK constituents in the stock and incubation solutions to the antisenescent effects were excluded, these *N*9-glucosides seem to exhibit the antisenescent activities per se.

### 3.4. Both N7- and N9-Glucosides of Trans-Zeatin Retain Chlorophyll in Tomato Leaf Discs

**Figure 4:**
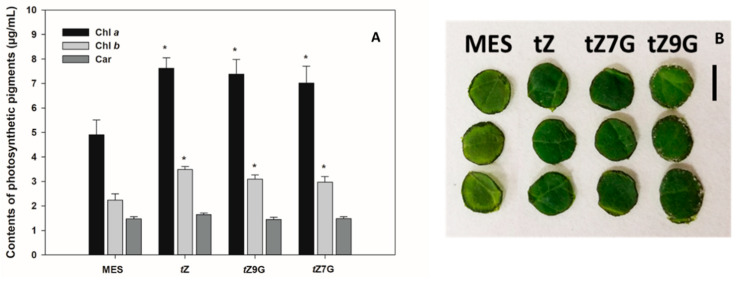
Senescence response to cytokinin *N*-glucosides in tomato leaf discs. Contents (µg·mL^−1^) of chlorophylls (Chl *a* and Chl *b*) and carotenoids (Car) were measured in tomato leaf discs incubated in the dark with MES with DMSO (MES buffer control) or 5 µM solutions of cytokinins (*t*Z, *t*Z7G and *t*Z9G) diluted in DMSO (**A**). Three replicates of tomato leaf discs after 6 d incubation in darkness with MES with DMSO (MES buffer control) or 5 µM solutions of cytokinins (*t*Z, *t*Z7G and *t*Z9G) diluted in DMSO (**B**). Data are presented as an average ± SE of 3–4 different biological replicates. Asterisks indicate a statistically significant difference between MES buffer control and treated samples according to two-tailed paired Student’s *t*-test: * *p* < 0.05. *t*Z = *trans*-zeatin; -7G = *N*7-glucoside; -9G = *N*9-glucoside; DMSO = dimethyl sulfoxide.

To determine a senescence response to CK glucoconjugates also in dicot species, contents of chlorophyll *a* (Chl *a*), chlorophyll *b* (Chl *b*) and carotenoids (Car) were analyzed in excised tomato leaf discs incubated for 6 d in the dark with MES (buffer control) or 5 µM solutions of *t*Z, *t*Z7G and *t*Z9G. Based on chlorophyll retention, strong antisenescent effects were recorded for *t*Z (7.62 µg·mL^−1^ Chl *a* and 3.48 µg·mL^−1^ Chl *b*) as well as for both its *N*-glucosides *t*Z7G (7.02 µg·mL^−1^ Chl *a* and 2.97 µg·mL^−1^ Chl *b*) and *t*Z9G (7.39 µg·mL^−1^ Chl *a* and 3.1 µg·mL^−1^ Chl *b*) as compared to the control (4.91 µg·mL^−1^ Chl *a* and 2.24 µg·mL^−1^ Chl *b*) (Figure 4A,B). No significant differences were found between control and any of the tested CKs in Car concentrations (Figure 4A).

These results indicate that not only *t*Z but also *t*Z7G and *t*Z9G retain chlorophyll in excised tomato leaf discs and thus considerably participate in the leaf senescence process in tomato. In general, our data also suggest that the antisenescent effects of exogenously applied CK *N*7- but not *N*9-glucosides may considerably differ in monocot and dicot plants (Figure 3 and Figure 4).

### 3.5. Metabolism and Accumulation of N7- and N9-Glucosides of Trans-Zeatin in Oat Leaf Segments

**Figure 5:**
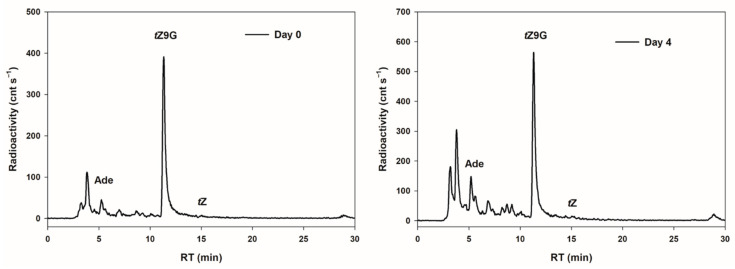
Metabolism of exogenously applied radiolabelled [2-^3^H]*trans*-zeatin 9-glucoside in oat leaf segments. The peaks represent the distribution of radioactivity associated with individual metabolites in oat leaves at time 0 and incubated for four (right) days in [2-^3^H]*trans*-zeatin *N*9-glucoside ([2-^3^H]*t*Z9G). The products of [2-^3^H]*t*Z9G metabolism were analysed by HPLC coupled to on-line radioactivity detector. *t*Z9G = *trans*-zeatin-9-glucoside (RT = 11.33 min); Ade = adenine (RT = 5.23 min); *t*Z = *trans*-zeatin (RT = 5.23 min).

In order to examine possible interconversions of *t*Z9G to *t*Z as a potential cause of its established bioactivity in the oat leaf chlorophyll retention assay, the metabolic fate of [2-^3^H]*t*Z9G was investigated in detached oat leaves. After 4 d incubation in darkness, the most significant proportion (45.7% of total metabolites) of [2-^3^H]*t*Z9G was retained in an unmetabolized form and no conversion to [2-^3^H]*t*Z was found. On the other hand, appreciable amounts of adenine (although not much higher than in the stock solution) and increasing peaks of other unidentified hydrophilic metabolites (possibly adenine *N*9-glucoside of which we did not have a standard at the time of the study) were detected in leaves following 4 d incubation (Figure 5).

**Figure 6:**
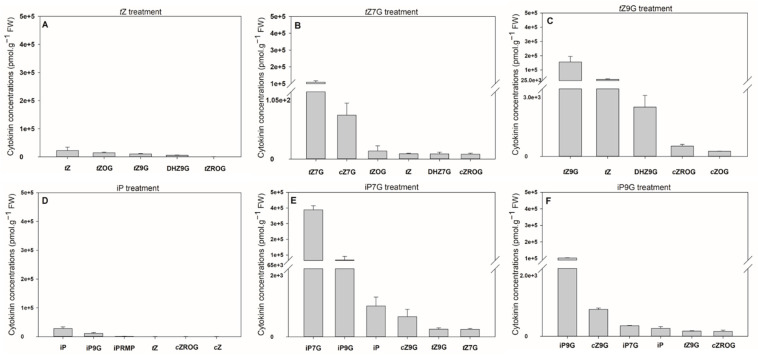
Endogenous cytokinin profiles in oat leaf segments following exogenous treatments with *trans*-zeatin, *N^6^*-(Δ^2^-isopentenyl)adenine and their *N*7- and *N*9-glucosides. Endogenous cytokinin (CK) concentrations (in pmol·g^−1^ FW) were measured in oat leaf segments incubated for four days at the darkness with 0.5 mM solution of *t*Z (**A**), *t*Z7G (**B**), *t*Z9G (**C**), iP (**D**), iP7G (**E**) and iP9G (**F**). The levels of CK derivatives in controls are presented in Supplementary Table S4. *t*Z = *trans*-zeatin; iP = *N*^6^-(Δ^2^-isopentenyl)adenine; -7G = *N*7-glucoside; -9G = *N*9-glucoside. Data are presented as mean ± standard error (SE) of three biological replicates.

With respect to the limited availability of [2-^3^H]*t*Z9G and the complete unavailability of other tritiated CK *N*-glucosides, accumulation and metabolic conversions of unlabeled *t*Z, *t*Z7G and *t*Z9G were assessed in detached oat leaf segments and compared with those of iP, iP7G and iP9G. These experiments revealed that the oat leaves accumulated CKs to a much higher degree after their treatment with *N*-glucosides compared with the treatment with corresponding free bases (up to 13.8-fold for iP7G vs. iP and 6.9-fold for *t*Z9G vs. *t*Z) (Figure 6, Supplemental Table S4). Following concentrations were observed after exogenous application of respective CKs: iP7G (387 714.5 pmol·g^−1^ FW) > *t*Z9G (155 535.7 pmol·g^−1^ FW) > *t*Z7G (108 463.5 pmol·g^−1^ FW) > iP9G (103 004.5 pmol·g^−1^ FW) > iP (28 127.8 pmol·g^−1^ FW) > *t*Z (22 463.8 pmol·g^−1^ FW). It demonstrates that exogenously applied CK *N*-glucosides increased contents of their respective endogenous counterparts from ca. 520-fold (iP9G) to ca. 1950-fold (iP7G) in comparison with C1 and C2 controls (both containing around 200 pmol·g^−1^ FW of total CK *N*-glucosides) (Figure 6E,F and Supplemental Table S4).

Some CK metabolites were detected in oat leaves after 4 d incubation with each CK (Figure 6, Supplemental Table S4). Interestingly, a considerable increase in endogenous *t*Z levels (enhanced up to 11,000-fold making up 22% of the total CKs) was found in some experiments following the amendment of *t*Z9G, which is in contrast with an only 40-fold enhancement of *t*Z following *t*Z7G treatment (Figure 6B,C, Supplemental Table S4). This finding holds only for *N*-glucosides of *t*Z since any similar difference was not found as a result of iP7G and iP9G treatments on the formation of iP (Figure 6E,F, Supplemental Table S4). As for the iP-type CKs, a relatively high concentration of iP9G was found in leaves incubated with both iP and iP7G (representing 26.6% and 14.9%, respectively, related to the total CK levels) in contrast to a very slight proportion of iP7G (0.3%) detected in samples treated by iP9G (Figure 6D,F, Supplemental Table S4). The increase in endogenous *t*Z following application of unlabeled *t*Z9G is in contradiction with the established metabolic stability of tritiated [2-^3^H]*t*Z9G demonstrated above in the same system. There is, however, no experimental evidence showing that *t*Z is formed as a direct product of unlabeled *t*Z9G hydrolysis and thus its origin from other CK derivatives cannot be excluded.

Taken together, applications of [2-^3^H]*t*Z9G did not reveal any conversion to *t*Z in oat leaves. Our data also demonstrate a high accumulation of *N*-glucosides of *t*Z and iP in comparison to their free bases in the oat leaf segments. Moreover, the CK profiles reveal substantial amounts of *t*Z in the leaves incubated with *t*Z9G in contrast with the *t*Z7G treatment.

### 3.6. Different Effects of Trans-Zeatin N7- and N9-Glucosides on Phenotype Traits and Gene Expression in Maize

CKs are well known to inhibit plant root growth (e.g., [4]). Thus, we observed the effects of *t*Z, *t*Z7G and *t*Z9G (applied in 0.1 µM and 5 µM solutions) on the root and primary leaf length in hydroponically cultivated maize plants.

**Figure 7:**
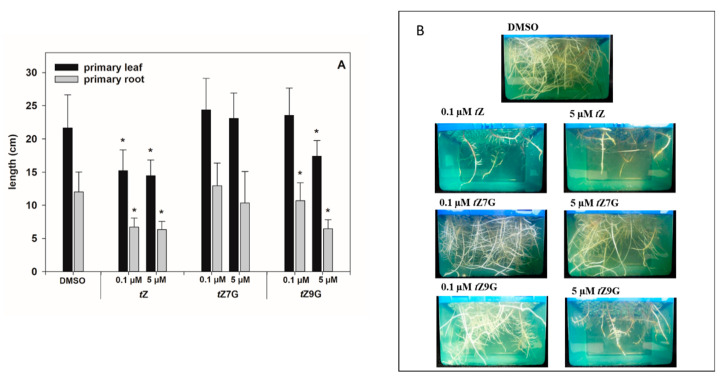
Effect of *trans*-zeatin and its *N*7- and *N*9-glucosides on maize phenotype traits. Length of primary leaves and roots (**A**) of maize plants cultivated hydroponically in 0.1 µM and 5 µM solutions of cytokinins (CKs) *t*Z, *t*Z7G and *t*Z9G. The effects of individual CK treatments on primary roots (**B**). *t*Z = *trans*-zeatin; -7G = *N*7-glucoside; -9G = *N*9-glucoside. Asterisks indicate a statistically significant decrease in length between the control (DMSO) and CK-treated plants according to two-tailed unpaired Student’s *t*-test: * *p* < 0.05.

As for the primary leaves, the length measurements in control plants (21.66 ± 4.97 cm) and CK treatments revealed that *t*Z treatment (0.1 µM and 5 µM) significantly decreased primary leaf growth (1.4- and 1.5-fold, respectively), which also holds for 5 µM *t*Z9G (1.3-fold; Figure 7A). In contrast, the application of 0.1 M *t*Z9G and *t*Z7G, as well as the amendment of 5 µM *t*Z7G, lacked the inhibitory effect on primary leaf growth in maize (Figure 7A).

As expected, plants grown on *t*Z exhibited significantly shorter roots (ca. 1.8–1.9-fold) compared to the control (12.02 ± 3.0 cm; Figure 7). In analogy, the addition of *t*Z9G in 5 µM concentration resulted in potent inhibition of maize root length (6.45 ± 1.38 cm), which was in contrast to *t*Z7G treatment (10.33 ± 4.76 cm) (Figure 7). When applied at a lower concentration, no inhibitory effects on maize growth were found for *t*Z7G (12.94 ± 3.41 cm), but statistically significant reduction of root length (1.1-fold) was recorded for *t*Z9G (Figure 7).

**Figure 8:**
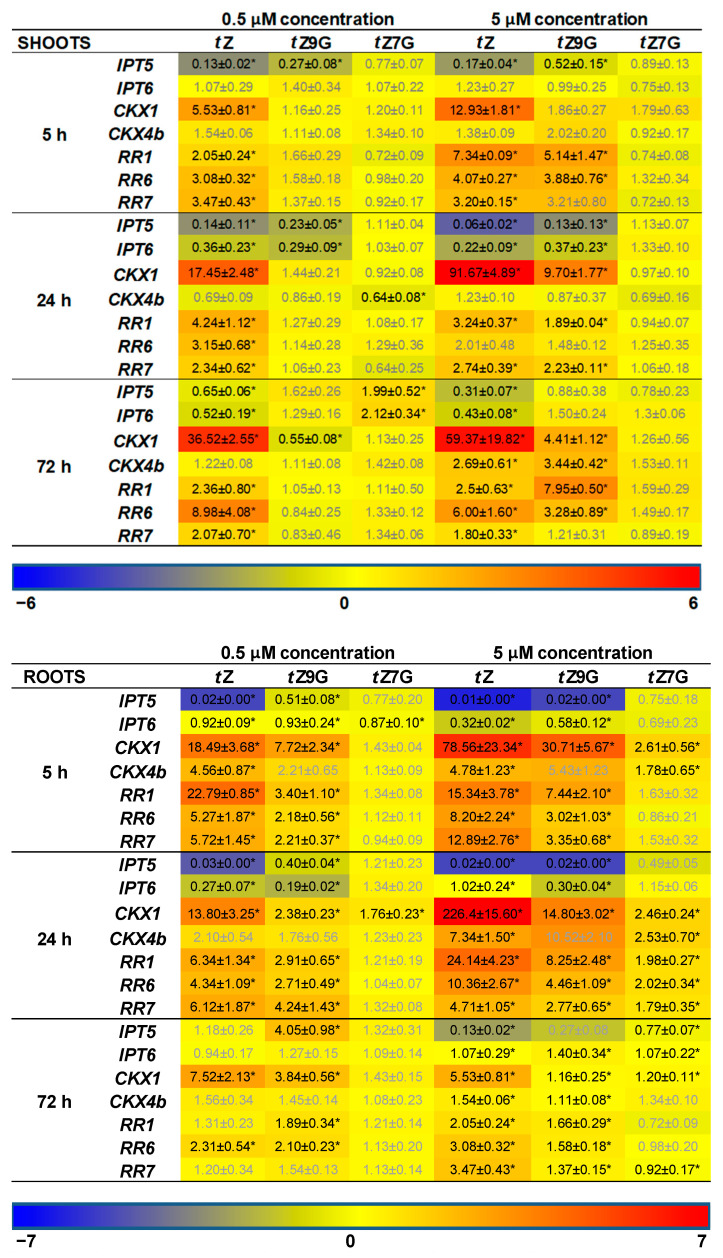
Effect of *trans*-zeatin and its *N*7- and *N*9-glucosides on selected cytokinin metabolic genes in maize leaves and roots. Heatmaps present the relative expression levels of selected cytokinin (CK) genes in leaves and roots of maize plants cultivated hydroponically in 0.5 µM and 5 µM solutions of CKs *t*Z, *t*Z7G and *t*Z9G for 5 h, 24 h and 72 h. Relative values from qRT-PCR were normalized separately for each plant tissue against results from control (methanol) conditions. Logarithmic values were converted into heatmaps using BAR HeatMapper Plus Tool (http://bar.utoronto.ca). *t*Z = *trans*-zeatin; -7G = *N*7-glucoside; -9G = *N*9-glucoside. Asterisks indicate a statistically significant difference between the control (methanol) and CK-treated samples according to two-tailed paired Student’s *t*-test: * *p* < 0.05.

In addition, CK-related gene expression profiles in maize plants hydroponically cultivated in 0.5 µM and 5 µM CK solutions were followed. Results of qRT-PCR showed more significant changes in relative gene expression profiles in roots than in leaves (Figure 8). Both of the applied *t*Z concentrations significantly upregulated the expression of most CK-related genes in roots, mainly after 5 h and 24 h, compared to the control. The most substantial upregulatory effects of both 0.5 µM and 5 µM *t*Z application were revealed for *ZmCKX1* expression that increased noticeably during 24 h and then slightly decreased with the length of action. Similarly, a relatively strong upregulation of *ZmRR1* was also found in roots after 5 h and 24 h treatments of the two *t*Z concentrations. Interestingly, both 0.5 µM and 5 µM *t*Z strongly downregulated *ZmIPT5* in the course of 24 h.

The addition of *t*Z9G caused a significant enhancement of mRNA transcriptional activity of some CK-related genes, mainly when applied at the higher concentration (5 µM) and acted for 5 h and 24 h, as shown for, e.g., *ZmCKX1* and *ZmRR*s (Figure 8). In analogy with the *t*Z treatment, application of 5 µM *t*Z9G significantly downregulated expression pattern of *ZmIPT5* during 24 h. Upregulation of *ZmCKX1* and *ZmRR*s was also elicited by 5 µM *t*Z7G during 24 h. However, the effect was much weaker than after the *t*Z9G treatment. The application of *t*Z9G in the lower concentration (0.5 µM) caused a few significant changes in the gene expression patterns, which primarily holds for the upregulation of *ZmCKX1* after 5 h and 72 h and *ZmRRs* after 5 h and 24 h. No statistically significant effects were recorded for 5 and 72 h treatments by 0.5 µM *t*Z7G with the only exceptions of *ZmIPT6* downregulated after 5 h and *ZmCKX1* upregulated after 24 h (Figure 8).

In leaves, a significant upregulation of expression profiles was found for most of the CK-related genes except for *ZmIPT*s upon incubation in 5 µM *t*Z and *t*Z9G solutions. In contrast, no statistically significant effects were recorded for *t*Z7G (Figure 8). Application of the lower concentration (0.5 µM) of both *N*-glucosides resulted only in a few substantial changes in the gene expression patterns in leaves, which primarily holds for the downregulation of *ZmIPT*s after 5 h and 24 h and *ZmCKXs* after 24 h and 72 h (Figure 8).

Altogether, the changes in maize root and leaf growth, as well as expression analysis, suggest that the applications of *t*Z7G and *t*Z9G provoke diverse effects on phenotype traits as well as on transcription levels of CK-related genes in maize plants.

### 3.7. Both Trans-Zeatin N7- and N9-Glucosides Induce Tomato TCSv2::3XVENUS Expression

**Figure 9:**
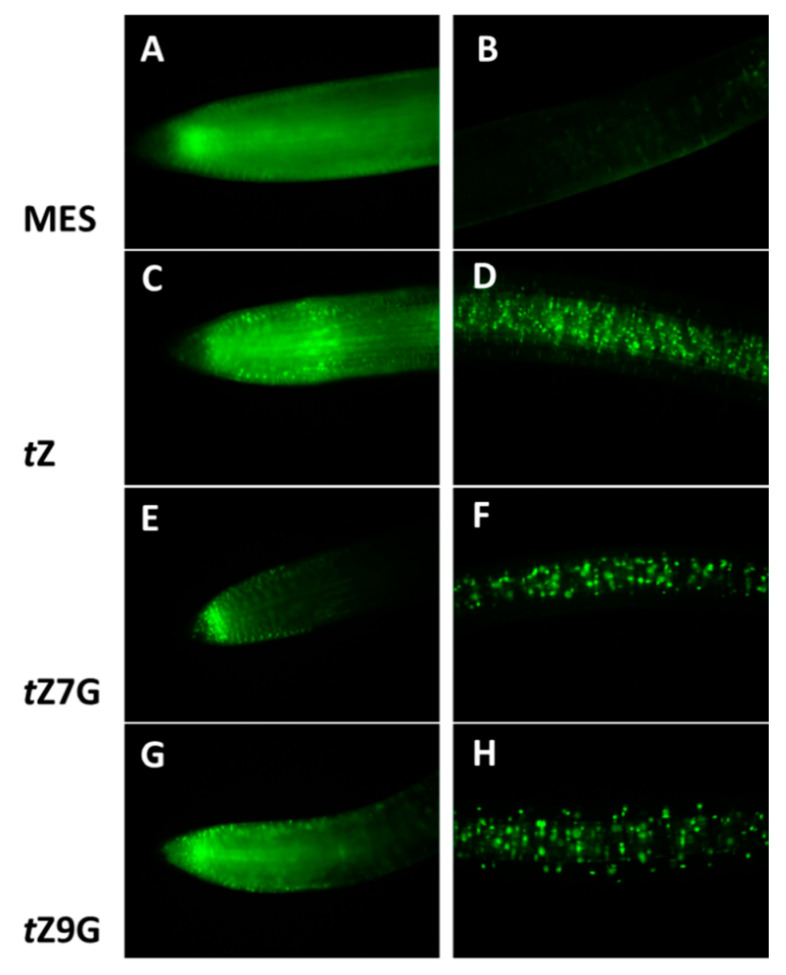
Effect of *trans*-zeatin and its *N*7- and *N*9-glucosides on cytokinin signal transduction pathway visualized by *TCSv2::3XVENUS* in tomato primary roots. The response of cytokinin (CK)-responsive reporter line *TCSv2::3XVENUS* to 5 µM *t*Z (**C**,**D**), *t*Z7G (**E**,**F**) and *t*Z9G (**G**,**H**) solutions. Tomato primary root tip (**A**,**C**,**E**,**G**) and the region approximately 1 cm back from the root tip (**B**,**D**,**F**,**H**) in comparison to the MES control (**A**,**B**) were examined for fluorescence activity. *t*Z = *trans*-zeatin; -7G = *N*7-glucoside; -9G = *N*9-glucoside.

To visualize a CK signal response to exogenous treatment with *t*Z, *t*Z7G and *t*Z9G, a cytokinin-responsive reporter line *TCSv2::3XVENUS* in tomato seedlings was analyzed after 24 h incubation in MES (control) and 5 µM solutions. The primary root tip and to the region approximately 1 cm back from the root tip were examined for fluorescence activity (Figure 9).

As expected, *t*Z notably induced *TCSv2::3XVENUS* expression in the root tip as well as in the central cylinder (Figure 9C,D) as compared to the control in which only an endogenous response was observed (Figure 9A,B). Surprisingly, the comparable fluorescence signal was induced by application of *t*Z9G (Figure 9G) in contrast to the *t*Z7G treatment enhancing the expression pattern only in the very end of the root tip, being arranged specifically in a stripe (Figure 9E). Both *t*Z7G and *t*Z9G also caused an increased activity at the distant area from the root tip, although to a lesser extent than *t*Z (Figure 9D,F,H).

To summarize, the expression of *TCSv2::3XVENUS* in tomato primary roots was specifically induced by exogenous *t*Z, *t*Z7G and *t*Z9G treatments suggesting that both *N*-glucosides are involved in transcriptional activation of CK signaling pathway visualized by a fluorescent reporter, but surprisingly the *N*-glucosides led to the activation of CK signaling in different parts of the root than *t*Z.

### 3.8. Effects of Trans-Zeatin N7- and N9-Glucosides in Cytokinin Receptor Activation Test

In order to unravel a potential activation of CK receptors by *t*Z7G or *t*Z9G, a receptor-ligand interaction was assayed in the receptor activation test using *E. coli* strain expressing *Arabidopsis* and maize *HKs*. Bacterial cultures incubated with *t*Z7G and *t*Z9G at different concentrations (from 0.001 to 0.5 µM) were investigated for the β-D-galactosidase activity. Neither *Arabidopsis* receptors, AHK3 and AHK4, nor maize receptors, ZmHK1 and ZmHK3, showed any significant response to *t*Z *N*-glucoside treatments throughout the whole concentration range (data not shown). Additionally, a possible interaction of *t*Z7G and *t*Z9G with the *Arabidopsis* and maize HK receptors was tested in the live-cell competitive binding assay. *E. coli* cells expressing *AHK3*, *AHK4*, *ZmHK1,* and *ZmHK3* receptors were incubated with either [2-^3^H]*t*Z alone or mixed with unlabeled CKs including *t*Z, *t*Z7G and *t*Z9G. While the unlabeled *t*Z (as well as other CK controls) inhibited the binding of [2-^3^H]*t*Z to HK receptors, *t*Z7G and *t*Z9G binding was comparable with adenine applied at the same concentration range and used as a negative control in all receptor tests (data not shown).

To generalize, these results revealed that no measurable CK-like activity of *t*Z7G and *t*Z9G could be linked to the tested *Arabidopsis* and maize HK receptors.

### 3.9. Trans-Zeatin N7- and N9-Glucosides are Not Substrates of Cytokinin Oxidase/Dehydrogenase but Induce Its Activity in Oat

The affinity of CKX from excised oat leaves towards the CK *N*-glucosides was investigated by testing the competitive effects of unlabeled CKs (2 µM and 20 µM) on the in vitro degradation of [2-^3^H]iP in the standard CKX assay (Table 1).

As expected, unlabeled iP was the most potent inhibitor of the degradation of the labeled substrate, followed by *t*Z. Both iP and *t*Z ribosides weakly inhibited the degradation when applied at a concentration of 20 µM, that is, 10-fold higher than that of a substrate. Indisputably, neither *N*7- nor *N*9-glucosides of iP or *t*Z inhibited the conversion of [2-^3^H]iP to adenine.

The effect of exogenous CK *N*-glucosides on CKX activity in vivo was measured in oat leaf segments incubated in 0.1 mM and 0.5 mM solutions and compared with fresh control leaves (C1) and water-treated control ones (C2). Whereas the initial CKX activity immediately after the excision of the segments (C1) was 0.196 nmol adenine per gram FW per hour, after 4 d incubation in the dark 11.4-fold enhancement was reached. All tested CKs, including *t*Z, iP, and their *N*7- and *N*9-glucosides, enhanced the effect even further (Table 2).

As expected, the highest increase in the CKX activity in oat leaves was recorded for *t*Z (up to 3.6-fold compared to C2) and iP (3.3-fold). Somewhat lower enhancement compared with the free bases was caused by *N*7-glucosides (up to three-fold *t*Z7G and 2.4-fold iP7G) followed by *N*9-glucosides (up to 2.1-fold *t*Z9G and 1.9-fold iP9G).

Taken together, although non-substrates of the oat leaf CKX, both externally applied *N*7- and *N*9-glucosides can induce the CKX activity in vivo.

## 4. Discussion

Simultaneously with enormous recent progress in CK research, many new questions related to metabolism and function(s) of particular CK forms arose. In this study, we focus on the characterization of *N*-glucosylation of endogenous CKs and the biological significance of its different *N*7- and *N*9-glucosides products.

Our comprehensive screening throughout the plant kingdom supports the hypothesis that CK *N*-glucoconjugates occur ubiquitously in vascular plants. However, their occurrence as well as the proportion between *N*7- and *N*9-glucosides are highly variable (Figure 1, Supplemental Table S1 and Figure S1). The schematic overview based on a set of one hundred species generally indicates an increasing trend in concentrations of CK *N*-glucosides from evolutionary older plants such as Horsetails and Ferns to the youngest clades represented by Superasterids and Asterids (Figure 1, Supplemental Table S1 and Figure S1). These data are in accord with the reported absence or deficiency of CK *N*-glucoconjugates in evolutionary older non-vascular plants including algae [47,69] and mosses [46] as well as in fungi [70,71]. The absence or low levels of CK-glucoconjugates together with a prevalence of *c*Z-type CKs in non-vascular plants [46,47,48] suggest that function(s) of missing or negligible CK *N*-glucosides might be fulfilled in evolutionary older species by *c*Z and its derivatives [72] and indicate a close interconnection between CK *N*-glucosyltransferase pathway and production of *c*Z-types in the evolutionary context. Despite the upward trend in the levels of total CK *N*-glucoconjugates throughout the evolution of the plant kingdom, no analogous parallel with evolutionary complexity is evident for the ratio between *N*7- and *N*9-glucosides.

CK *N*-glucosides have been for years considered irreversibly inactive or weakly active CK forms (e.g., [3]) unrecognized by bacterial and *Arabidopsis* CK receptors [7,9,20] and accumulating mainly in the vacuoles and outside the plant cells [49]. In agreement with work of Šmehilová et al. [20], our data show that the levels of total CK *N*-glucoconjugates are dramatically increasing in both leaves and roots throughout the life span of *Arabidopsis* used as a model plant (Figure 2A,B). This increase is primarily due to the rising concentrations of the predominant *N*7-glucosides, iP7G and *t*Z7G (Figure 2C,D; [16,18,32]). Although the generally accepted hypothesis that *N*-glucosylation irreversibly inactivates CKs has been recently questioned [32] the progressive accumulation of leaf and root CK *N*-glucosides throughout *Arabidopsis* ontogenesis supports them to be regarded as final products of CK metabolism [73]. This holds true especially for *N*7-glucosides which accumulate predominantly during the *Arabidopsis* growth this work and [20] and are not degraded by CKX, while *N*9-glucosides are often preferred substrates of some CKX isozymes (e.g., [42,74]) and do not accumulate to such extent.

The increasing accumulation of CK *N*-glucosides during *Arabidopsis* ontogenesis is not connected with the same tendency in *UGT76C1* and *UGT76C2* gene expression patterns. RT-qPCR using RNA from *Arabidopsis* leaves and roots revealed different dynamics of expression levels of the two genes (Figure 2E,F). A considerably higher expression of *UGT76C2* compared to the *UGT76C1* transcripts in both leaves and roots indicates its crucial role in the formation of CK *N*-glucosides. It corresponds well with the higher physiological impact of UGT76C2 reported by other authors [16,18,19].

The apparent inconsistency between the rate of increase of CK *N*-glucosides and dynamics in *UGT76C1* and *UGT76C2* gene expression levels throughout *Arabidopsis* ontogenesis indicates the existence of other mechanisms apart from the UGT pathway regulating the levels of *N*7- and *N*9-glucosides and their homeostasis in plants such as, e.g., downregulation of CK *N*9-glucosides by some CKX isozymes oppositely to CK *N*7-glucosides which are not degraded by CKXs and accumulating mainly in Arabidopsis leaves (Figure 2C,D).

Physiological significance of CK *N*7- and *N*9-glucosides was evaluated based on their impact on both oat and tomato leaf chlorophyll retention, as well as maize root growth inhibition. When applied at high concentrations (0.1 and 0.5 mM), *t*Z9G and DHZ9G suppressed the chlorophyll degradation in detached oat leaf pieces (Figure 3). The suppression was, however, not demonstrated for their corresponding *N*7-counterparts (Figure 3). As neither [2-^3^H]*t*Z9G → [2-^3^H]*t*Z conversion (Figure 5) nor any affinity of β-D-glucosidase to *t*Z9G (Supplemental Figure S4) were observed, the *N*9-glucosides are supposed to exert their antisenescent activities differently than via hydrolysis to free *t*Z form. This assumption is also supported by the *t*Z9G metabolic stability in the stock and incubation solutions (Supplemental Figure S3 and Table S3). In contrast to oat leaves, not only *t*Z9G but also *t*Z7G was shown to retain chlorophyll in tomato leaf discs (Figure 4) and *Arabidopsis* detached cotyledons [28]. It indicates possible roles of CK *N*-glucosides in the senescence process in the dicotyledonous plants as well. The antisenescent impact of CK *N*-glucosides agrees with previous reports that an essentially intact adenine nucleus in the CK molecule is required for high growth-promoting effects, but a substitution by one atom for another might lead to the CK activity as well in e.g., senescence assay [75]. The diversity between the used models (oat leaves vs. tomato leaves and *Arabidopsis* cotyledons) indicates that the antisenescent effects of exogenous CK *N*9- and particularly *N*7-glucosides may differ in relation to their phylogenetic relationships to each other and that separate mechanisms of their action cannot be ruled out between monocots and dicots and/or between *N*9- and *N*7-glucosides.

Our results substantially question putative biological inactivity of CK *N-*glucoconjugates. They correspond well with very recently demonstrated biological effects of *t*Z *N*7- and *N*9-glucosides and their impact on the transcriptome and proteome in *Arabidopsis* [28]. Additionally, a biological activity (although very low and insignificant) of *tZ*9G on chlorophyll retention was reported previously in detached wheat leaves [23]. The inability of iP and its *N*7- and *N*9-glucosides to inhibit the dark-induced senescence (Supplemental Figure S2) is in accord with a very low sensitivity of chlorophyll retention bioassays generally known for iP-type CKs [76,77,78]. It also indicates distinct properties and metabolic conversions of *t*Z- and iP-type CKs as suggested by Hošek et al. [32].

Also, the established metabolic stability of [2-^3^H]*t*Z9G in oat leaves agrees with the results reported by Hallmark et al. [28] in *Arabidopsis*. Although no interaction of CK *N*-glucosides with the CK perception system has been reported yet, it is suggested that *t*Z9G exhibits the antisenescent activity in the oat leaves per se. To support this suggestion, numerous data are showing that the substitution of hydrogen by various substituents at the *N*9-position of the CK purine ring is not necessarily associated with the loss of biological activity [30,33,78,79]. On the other hand, this is in contrast to the data demonstrating immediate conversion to *t*Z in *Arabidopsis* after treatments with unlabeled *t*Z7G and *t*Z9G as well as [2-^3^H]*t*Z9G [32]. Similar to these findings, Podlešáková et al. [33] reported an increase of free CKs, DHZ and BA, in maize tissues after treatments with their tritium-labeled *N*9-glucosides. Moreover, contrary to our results showing the inability of oat β-D-glucosidase to hydrolyze *t*Z9G (Supplemental Figure S4) the recombinant maize β-D-glucosidase Zm-p.60.1 was found to cleave *t*Z9G but not *t*Z7G in vitro [37]. The discrepancy in the existing data from the CK *N*-glucoside metabolic studies might be possibly due to distinct experimental settings, the use of either labeled or unlabeled CK *N*-glucosides of different provenience, various plant materials (monocots vs. dicots), etc. In contrast to the demonstrated metabolic stability of [2-^3^H]*t*Z9G in oat leaves, an efficient accumulation of exogenously applied unlabeled *t*Z9G was associated with an increase in endogenous *t*Z in some of our experiments. This finding makes the story even more complicated. However, there is no experimental evidence for *t*Z9G hydrolysis, thus excluding *t*Z origin by other means.

CKs are generally known as natural inhibitors of the plant root growth (e.g., [4,80,81]). However, data regarding the effects of CK *N*-glucosides on the roots are still rather rare and show no inhibition by *N*7- and *N*9-glucosides of *t*Z and BA [4,28,82]. In this work, analogously to the antisenescent effects in oat leaves, potent inhibition of the maize root length was found for *t*Z9G but not *t*Z7G (Figure 7). The impact on leaf growth was less pronounced, and thus only the high concentration of *t*Z9G showed an effect (Figure 7). A summary of biological effects displayed by CK *N*7- and *N*9-glucosides in various plant systems as found in this study and recently published [28] is shown in Table 3. The presented data indicate that the CK *N*-glucosides display distinct biological effects in various plant models, mostly as antisenescence compounds though.

Different responses of mRNA transcript levels of CK-related genes to *t*Z7G and *t*Z9G treatments were revealed in maize (Figure 8). With a few exceptions, a more substantial upregulatory effect of *t*Z9G which in general copied the response to *t*Z, was found on *ZmIPT, ZmCKX,* and *ZmRR* genes in both leaves and roots whereas *t*Z7G regulated most genes in the roots only when the higher concentration (5 µM) was applied (Figure 8). In general, roots are apparently more sensitive to changes in gene expression evoked by *t*Z7G and *t*Z9G than the leaves. This may be because of smaller outcomes of metabolic conversions and lack of transport, while roots reflect direct action of the glucosides. Additionally to the effects of *t*Z *N*7- and *N*9-glucosides on the CK-related genes expression in maize, both *t*Z7G and *t*Z9G induced the expression of *TCSv2::3XVENUS* signal in primary tomato root indicating their involvement in transcriptional activation of CK signaling pathway visualized by this reporter (Figure 9).

Hallmark et al. [28] suggested that *N*7- and *N*9-glucosides tend to affect the CK-related genes in Arabidopsis in a manner differing from *t*Z as well as from each other, despite there are some exceptions to this generalization. Although our data suggest interaction of *t*Z7G and *t*Z9G with CK response regulators in maize (Figure 8) and the expression of *TCSv2::3XVENUS* signal was visualized in transgenic tomato seedlings (Figure 9), direct interaction of *t*Z7G and *t*Z9G with *Arabidopsis* or maize cytokinin HK receptors could not be confirmed by the bacterial CK receptor assay and the live-cell binding assay using radiolabeled *t*Z. Together with our results from live-cell binding assay, this agrees with previous findings [7,9] showing that the *t*Z7G and *t*Z9G are not able to interact with the plant histidine kinase receptors. The core of the mechanisms or interactions causing responses in CK signaling pathways in plants after the CK *N*-glucosides treatments, however, remains to be clarified.

Based on the in vitro substrate competition experiments, the affinity of CKX from excised oat leaves towards the CK *N*-glucosides was determined. None of the tested glucosides (*t*Z7G, *t*Z9G, iP7G, iP9G) was revealed to inhibit the conversion of [2-^3^H]iP to adenine (Table 1) even though some recombinant AtCKX [42,43], barley HvCKX [44] and maize ZmCKX [45] enzyme isoforms show the ability to degrade CK *N*9-glucosides (but not CK *N*7-glucosides). Although not being substrates of oat CKX, the glucosides were effective in inducing the CKX activity in oat leaves in planta (Table 2). This indicates that their effect on CKX activity is not mediated by a simple induction of the enzyme by its substrates and corresponds with the previously reported enhancement of the CKX activity in senescing barley leaves [83] and tobacco calli [61,84]. The CK *N*-glucosides might be thus involved in a positive feedback mechanism of re-establishment and maintenance of CK homeostasis required for the development of physiological events such as chlorophyll retention and root growth as proposed by Kamínek et al. [85]. This autoinductive mechanism might represent one of the possible explanations of the biological effects of CK *N*-glucosides. However, it does not explain different actions between *N*7- and *N*9-glucosides in some of the bioassays.

## 5. Conclusions

Our findings argue against the general image of CK *N*-glucosides as irreversible CK forms lacking biological activities. We report here (1) a widespread distribution of CK *N*7- and *N*9-glucosides across the plant kingdom with distinct representation in the total CK pool increasing from lower (evolutionary older) to higher (evolutionary younger) plants, (2) their changing levels as well as the expression of *UGT76C1* and *UGT76C2* genes during *Arabidopsis* ontogenesis, (3) visible physiological activities in selected CK bioassays, (4) noticeable effects on expression of CK-related genes in maize and activation of *TCSv2::3XVENUS* signal in tomato, and (5) a role in inducing CKX activity in oats as an inherent part of autoinduction of CK-initiated physiological processes. Based on our results and very recent literature data [28,32], CK *N*-glucosides seem to be more prevalent and more relevant to CK biology being involved in CK evolution and having some unique function(s) in plants. Thus, the involvement of CK *N*-glucosides in the evolution and biology of CKs suggests that these long-ignored compounds merit more investigation, raising further questions about their physiological importance and mechanism of action in plants.

## Figures and Tables

**Table 1:** Substrate specificity of cytokinin oxidase/dehydrogenase in oat leaf segments. The substrate specificity of cytokinin oxidase/dehydrogenase (CKX) was determined based on the competitive effects of unlabelled cytokinins (CKs) on the in vitro degradation of [2-^3^H]iP in the standard CKX assay. Enzyme preparations were extracted and partially purified from oat leaves incubated four days at the darkness in water. The unlabelled CKs were applied in four replicates (*n* = 4) in vitro at 2 µM and 20 µM concentrations. Enzyme activity in control (water-treated oat leaf segments kept for four days in the dark) assayed without unlabelled CK was 2.989 ± 0.102 nmol Ade.g^−1^ FW.h^−1^ (=100%). iP = *N*^6^-(Δ^2^-isopentenyl)adenine; iPR = *N*^6^-(Δ^2^-isopentenyl)adenosine; *t*Z = *trans*-zeatin; *t*ZR = *trans*-zeatin 9-riboside; -7G = *N*7-glucoside; -9G = *N*9-glucoside; MP = -5′-monophosphate.

Concentration of Unlabeled Cytokinin	2 µM	20 µM
	CKX Activity (% of Control)	
iP	49.8 ± 3.3	10.3 ± 0.2
adenine	106.8 ± 1.0	98.3 ± 3.9
iPR	99.4 ± 5.4	60.8 ± 1.6
iP7G	104.4 ± 1.4	108.5 ± 1.4
iP9G	107.6 ± 4.5	104.8 ± 1.6
iPRMP	105.6 ± 6.8	86.7 ± 13.6
*t*Z	93.9 ± 0.3	26.5 ± 3.9
*t*ZR	97.5 ± 0.6	87.8 ± 3.2
*t*Z7G	107.7 ± 2.6	103.9 ± 2.5
*t*Z9G	99.6 ± 10.1	106.4 ± 0.2
*t*ZRMP	100.0 ± 8.0	106.6 ± 3.7

**Table 2:** Effect of *trans*-zeatin, *N*^6^-(Δ^2^-isopentenyl)adenine and their *N*7- and *N*9-glucosides applied exogenously to oat leaf segments on cytokinin oxidase/dehydrogenase activity. Cytokinin oxidase/dehydrogenase (CKX) preparations were extracted and partially purified from oat leaves incubated four days at the darkness in water or in tested cytokinin (CK) solutions (0.1 mM and 0.5 mM). The CKX activity was assayed as described in Material and Methods and compared with that in freshly harvested oat leaf segments before incubation (initial control C1) and in water-treated oat leaf segments kept for four days in the dark (water control C2). The results (the means of three biological replicates) are expressed as an increase in CKX activity (-fold) compared to C1 (0.196 ± 0.102 nmol Ade.g^−1^ FW.h^−1^) and C2 (2.238 ± 0.091 nmol Ade.g^−1^ FW.h^−1^). *t*Z = *trans*-zeatin; iP = *N*^6^-(Δ^2^-isopentenyl)adenine; -7G = *N*7-glucoside; -9G = *N*9-glucoside.

	Increase in CKX Activity (-fold)	
	(Compared to C1)	(Compared to C2)
C1	---	---
C2	11.4 ± 1.4	---
		
*t*Z (0.1 mM)	38.6 ± 2.7	3.4 ± 0.2
*t*Z (0.5 mM)	41.2 ± 3.4	3.6 ± 0.3
*t*Z7G (0.1 mM)	34.1 ± 5.0	3.0 ± 0.4
*t*Z7G (0.5 mM)	34.2 ± 5.5	3.0 ± 0.5
*t*Z9G (0.1 mM)	19.1 ± 3.2	1.7 ± 0.3
*t*Z9G (0.5 mM)	24.4 ± 1.3	2.1 ± 0.1
		
iP (0.1 mM)	37.2 ± 2.2	3.3 ± 0.2
iP (0.5 mM)	38.0 ± 3.0	3.3 ± 0.3
iP7G (0.1 mM)	27.5 ± 1.7	2.4 ± 0.2
iP7G (0.5 mM)	25.8 ± 3.6	2.3 ± 0.3
iP9G (0.1 mM)	22.0 ± 1.2	1.9 ± 0.1
iP9G (0.5 mM)	18.3 ± 1.1	1.6 ± 0.1

**Table 3:** Cytokinin *N*-glucosides (-like) activity in different monocot and dicot species. The table represents a summary of our data, part of which has already been published by Hallmark et al. [28]. *t*Z = *trans*-zeatin; DHZ = dihydrozeatin; iP = *N*^6^-(Δ^2^-isopentenyl)adenine; -7G = *N*7-glucoside; -9G = *N*9-glucoside.

	CK(-Like) Activity	Non-Active
oat	*t*Z	iP
detached first leaf	*t*Z9G DHZ9G	iP9G
senescence delay		iP7G *t*Z7G DHZ7G
tomato	*t*Z	
leaf discs	*t*Z9G	
senescence delay	tZ7G	
Arabidopsis	*t*Z	
excised cotyledons	*t*Z9G	
senescence delay	*t*Z7G	
maize	*t*Z	
root	*t*Z9G	
growth inhibition		*t*Z7G
Arabidopsis	iP *t*Z	
root		iP9G *t*Z9G
growth inhibition		iP7G *t*Z7G

## Supplementary Materials

The following are available online at https://www.mdpi.com/2218-273X/11/1/24/s1. Table S1: Complete list of plant species selected for the screen and concentrations of individual cytokinin (CK) *N*-glucosides, total CK *O*-glucosides and total CKs (in pmol·g^−1^ FW). *t*Z = *trans*-zeatin; DHZ = dihydrozeatin; iP = *N*^6^-(Δ^2^-isopentenyl)adenine; *c*Z = *cis*-zeatin; -7G = *N*7-glucoside; -9G = *N*9-glucoside. Data are presented as mean ± standard error (SE) of two independent measurements. Table S2: Concentrations of individual cytokinin *N*-glucosides (in pmol g^−1^ FW) in *Arabidopsis thaliana* leaves and roots during ontogeny. DAS = days after sowing; *t*Z = *trans*-zeatin; *c*Z = *cis*-zeatin; DHZ = dihydrozeatin; iP = *N*^6^-(Δ^2^-isopentenyl)adenine; -7G = *N*7-glucoside; -9G = *N*9-glucoside. Data are presented as mean ± standard error (SE) of three biological replicates. Table S3: Metabolic stability of *trans*-zeatin *N*7- and *N*9-glucosides in tested stock solutions used in the chlorophyll retention bioassay. Contents (in pmol.µL^−1^) of cytokinins (CKs) were measured in 1 µL of 0.1 mM *t*Z7G and *t*Z9G fresh stocks and incubation solutions with or without oat leaf segments after four days as described in Material and Methods. *t*Z = *trans*-zeatin; *c*Z = *cis*-zeatin; DHZ = dihydrozeatin; iP = *N*^6^-(Δ^2^-isopentenyl)adenine; -7G = *N*7-glucoside; -9G = *N*9-glucoside. Data represent mean ± standard error (SE) of two replicates. Table S4: Endogenous cytokinin levels (in pmol·g^−1^ FW) in oat leaf segments incubated for four days with 0.5 mM solutions of iP, iP7G, iP9G, *t*Z, *t*Z7G and *t*Z9G. C1—initial control (fresh leaves before incubation), C2—water control (excised oat leaves incubated for four days in the dark with water). *t*Z = *trans*-zeatin; *t*ZR = *trans*-zeatin 9-riboside; iP = *N*^6^-(Δ^2^-isopentenyl)adenine; iPR = *N*^6^-(Δ^2^-isopentenyl)adenosine; *c*Z = *cis*-zeatin; *c*ZR = *cis*-zeatin 9-riboside; DHZ = dihydrozeatin; DHZR = dihydrozeatin 9-riboside; -7G = *N*7-glucoside; -9G = *N*9-glucoside; -OG = *O*-glucoside; MP = -5′-monophosphate. Data are presented as mean ± standard error (SE) of three biological replicates. Figure S1: Occurrence and distribution of cytokinin (CK) *N*-glucosides (A), CK *N*9-glucosides (B), CK *N*7-glucosides (C) and CK *O*-glucosides (D) (expressed as % of total CKs) in selected main groups of vascular plants. Black dots represent the distribution of analyzed CK derivatives in individual plant species, blue and red triangles represent median and average values of all species within individual clades. Figure S2: Treatments with *N*^6^-(Δ^2^-isopentenyl)adenine (iP) and its *N*7- and *N*9-glucosides do not sustain chlorophyll content in oat leaf segments. Chlorophyll concentration is expressed as a percentage of the initial chlorophyll content of control fresh leaves before incubation (C1). Excised oat leaf pieces were incubated for 4 days in the dark with water (water control, C2) or solutions of cytokinins iP, iP7G and iP9G. Data are presented as mean ± SE of three biological replicates. Figure S3: Chemical stability (purity) of *trans*-zeatin *N*7- and *N*9-glucosides stocks. The top graph shows HPLC-MS data for internal standards ([^2^H_5_]*t*Z7G, [^2^H_5_]*t*Z9G). The lower two graphs demonstrate results for stocks of *t*Z7G (A) and *t*Z9G (B), respectively, used in the chlorophyll retention bioassay. Figure S4: Activity of β-D-glucosidase from oat leaves is not responsible for *trans*-zeatin *N*9-glucoside hydrolysis. The involvement of β-D-glucosidase activity from oat leaves in reactions with two substrates, *trans*-zeatin *O*-glucoside (*t*ZOG; A) and *trans*-zeatin *N*9-glucoside (*t*Z9G; B), both applied at 9 mM concentrations. Protein extract was prepared from 10 d old oat leaves treated with *t*Z9G (0.02 mM). A: 1—negative control (oat protein extract incubated in 0.05 M citrate-phosphate buffer, pH 5.5 (C-P buffer), for 96 h); 2—oat protein extract with *t*ZOG (9 mM) at 0 h; 3—oat protein extract incubated with *t*ZOG (9 mM) for 72 h; 4—oat protein extract incubated with *t*ZOG (9 mM) for 96 h; 5—*t*ZOG (9 mM in C-P buffer); 6—*t*Z (9 mM in DMSO). All samples and negative control were incubated at 37 °C, *t*ZOG standard was incubated for 96 h at 30 °C. Loading volume—2 µL. B: 1—negative control (oat protein extract incubated in 0.05 M C-P buffer for 96 h); 2—oat protein extract with *t*Z9G (9 mM) at 0 h; 3—oat protein extract incubated with *t*Z9G (9 mM) for 24 h, 4—oat protein extract incubated with *t*Z9G (9 mM) for 48 h; 5—oat protein extract incubated with *t*Z9G (9 mM) for 72 h; 6—oat protein extract incubated with *t*Z9G (9 mM) for 168 h, 7—*t*Z9G (9 mM in C-P buffer); 8—*t*Z (9 mM in DMSO). All samples and negative control were incubated at 37 °C, *t*Z9G standard was incubated for 168 h at 30 °C. Loading volume—2 µL.
