# Supplementary material for: Cytokinin N-glucosides: Occurrence, Metabolism and Biological Activities in Plants
Source: Biomolecules. 2020 Dec 28;11(1):24. doi: 10.3390/biom11010024 (PMC7824008; doi:10.3390/biom11010024)
Supplement: Supplementary file 1 [file biomolecules-11-00024-s001.zip › biomolecules-1036530-supplementary-1/Supporting figures 1-4.docx]

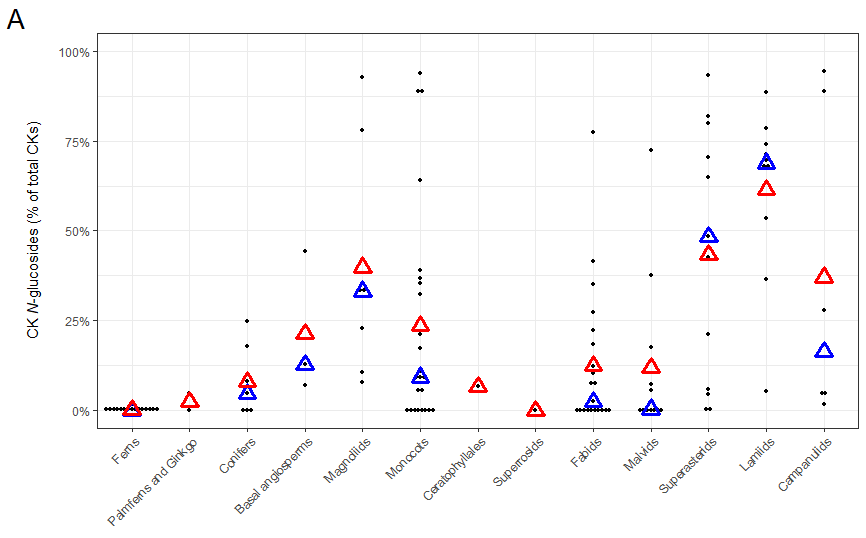

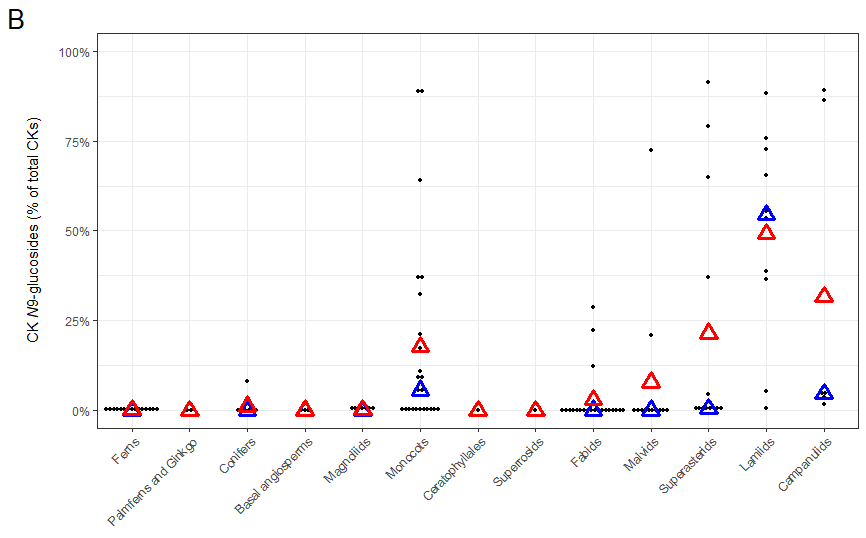

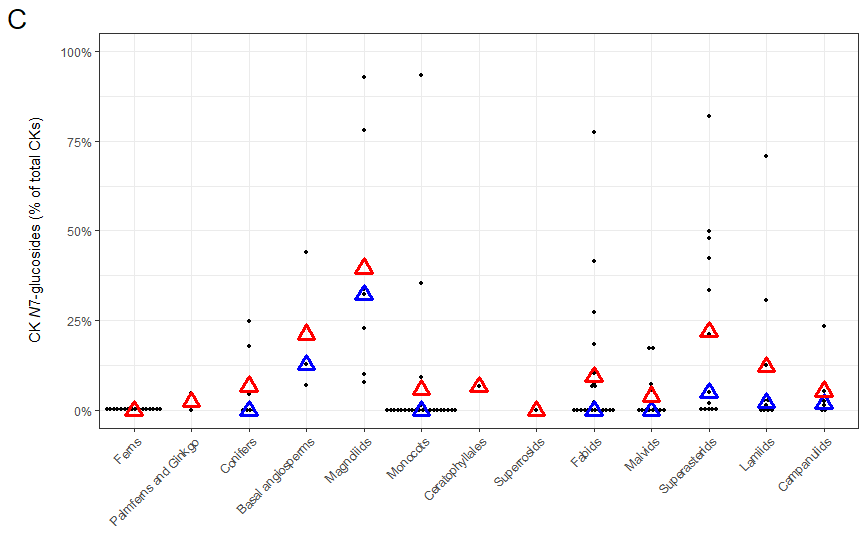

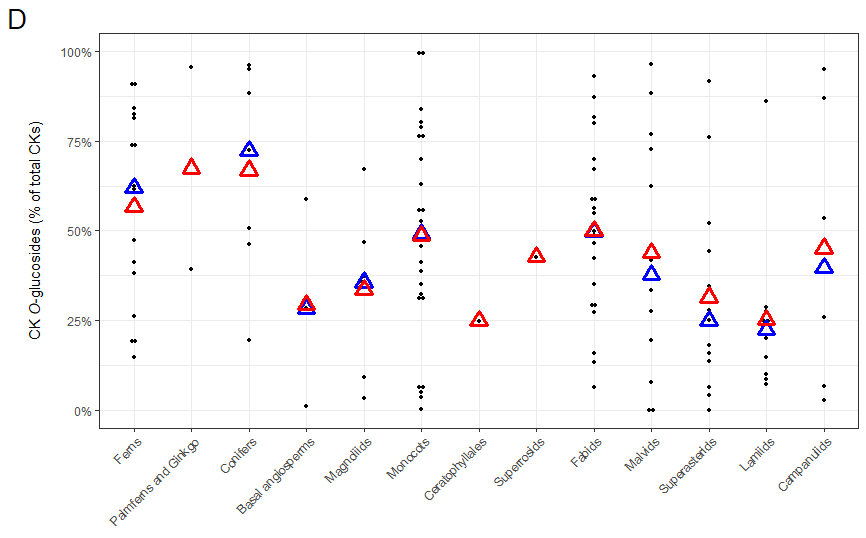


**Figure S1: Occurrence and distribution of cytokinin (CK) *N*-glucosides (A), CK *N9*-glucosides (B), CK *N7*-glucosides (C) and CK *O*-glucosides (D) (expressed as % of total CKs) in selected main groups of vascular plants**.

Black dots represent the distribution of analysed CK derivatives in individual plant species, blue and red triangles represent median and average values of all species within individual clades.


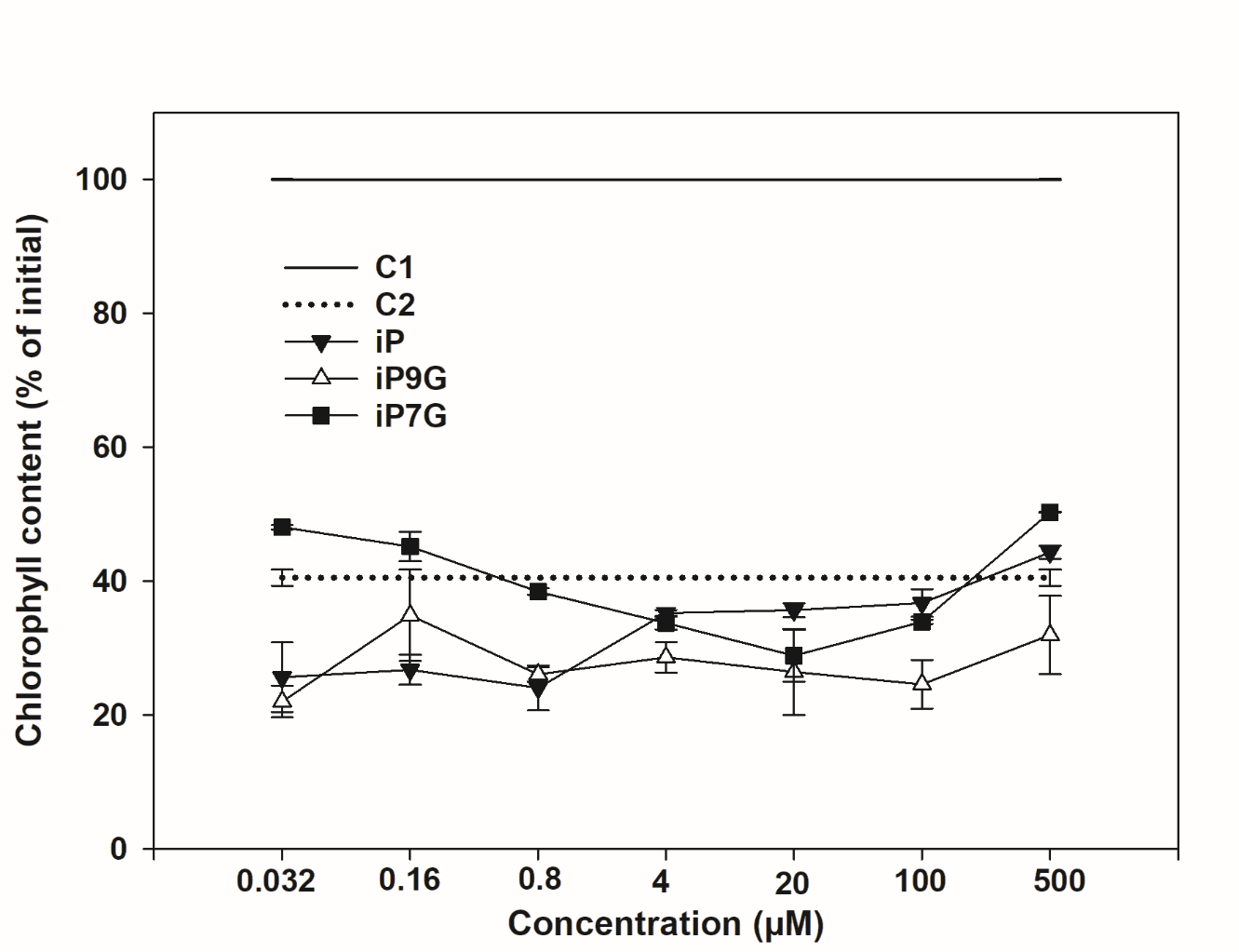


**Figure S2: Treatments with *N*^6^‑(Δ^2^‑isopentenyl)adenine (iP) and its *N*7- and *N*9-glucosides do not sustain chlorophyll content in oat leaf segments.**

Chlorophyll concentration is expressed as a percentage of the initial chlorophyll content of control fresh leaves before incubation (C1). Excised oat leaf pieces were incubated for 4 days in the dark with water (water control, C2) or solutions of cytokinins iP, iP7G and iP9G.

Data are presented as mean ± SE of three biological replicates.

**Figure S3: Chemical stability (purity) of** ***trans-*zeatin *N*7- and *N*9-glucosides stocks.**

The top graph shows HPLC-MS data for internal standards ([^2^H_5_]*t*Z7G, [^2^H_5_]*t*Z9G). The two peaks at RT = 12.5 min represent deuterated *zeatin*-*O*-glucoside (*ZOG*-d5) and dihydrozeatin (DHZ-d3) at RT = 13.28 min. The lower two graphs demonstrate results for stocks of *t*Z7G (A) and *t*Z9G (B), respectively, used in the chlorophyll retention bioassay. The chromatograms are TIC records of fragments m/z 387->207, 225 for internal standards and m/z 382->136, 148, 202, 220 for *zeatin*-glucosides.


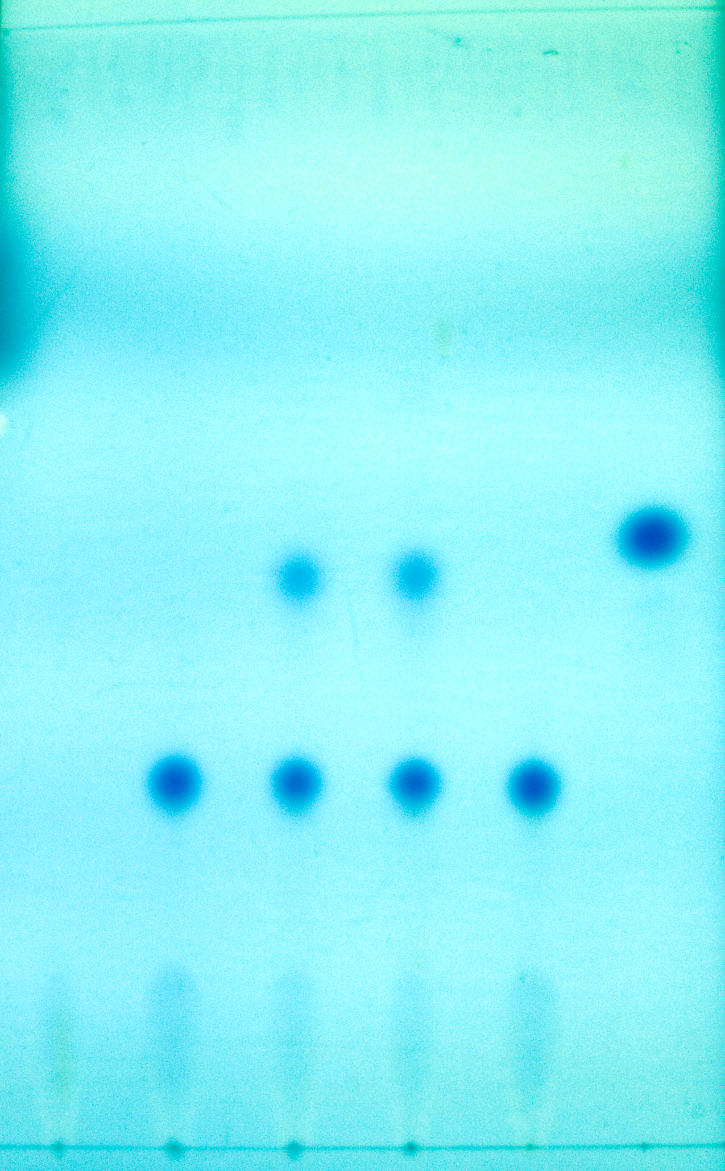


**1 2 3 4 5 6**

**A**

***t*Z**

***t*ZOG**


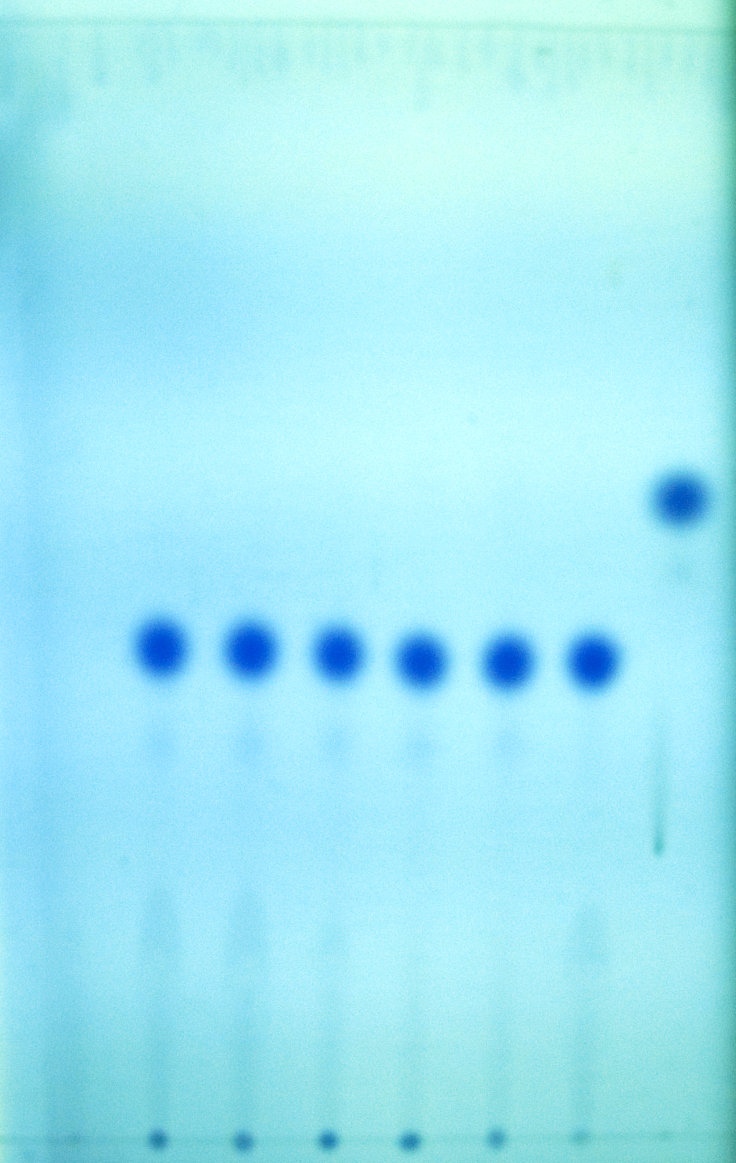


**1 2 3 4 5 6 7 8**

**B**

***t*Z**

***t*Z*9*G**

**Figure S4:** Activity of β-D-glucosidase from oat leaves is not responsible for *trans-*zeatin *N*9-glucoside hydrolysis. The involvement of β-D-glucosidase activity from oat leaves in reactions with two substrates, *trans-*zeatin *O*-glucoside (*t*ZOG; A) and *trans-*zeatin *N*9-glucoside (*t*Z9G; B), both applied at 9 mM concentrations. Protein extract was prepared from 10 d old oat leaves treated with *t*Z9G (20 µM). (A) 1 - negative control (oat protein extract incubated in 0.05 M citrate-phosphate buffer, pH 5.5 (C-P buffer), for 96 h); 2 - oat protein extract with *t*ZOG (9 mM) at 0 h; 3 - oat protein extract incubated with *t*ZOG (9 mM) for 72 h; 4 - oat protein extract incubated with *t*ZOG (9 mM) for 96 h; 5 - *t*ZOG (9 mM in C-P buffer); 6 – *t*Z (9 mM in DMSO). All samples and negative control were incubated at 37°C, *t*ZOG standard was incubated for 96 h at 30°C . Loading volume - 2 µL. (B) 1 - negative control (oat protein extract incubated in 0.05 M C-P buffer for 96 h); 2 - oat protein extract with *t*Z9G (9 mM) at 0 h; 3 - oat protein extract incubated with *t*Z9G (9 mM) for 24 h, 4 - oat protein extract incubated with *t*Z9G (9 mM) for 48 h; 5 - oat protein extract incubated with *t*Z9G (9 mM) for 72 h; 6 - oat protein extract incubated with *t*Z9G (9 mM) for 168 h, 7 - *t*Z9G (9 mM in C-P buffer); 8 – *t*Z (9 mM in DMSO). All samples and negative control were incubated at 37°C, *t*Z9G standard was incubated for 168 h at 30°C . Loading volume - 2 µL.
